# Simulated Annealing for Model‐Robust Partial Profile Choice Designs in Healthcare Preference Studies

**DOI:** 10.1002/sim.70770

**Published:** 2026-10-13

**Authors:** Yicheng Mao, Roselinde Kessels

**Affiliations:** ^1^ Department of Data Analytics and Digitalization Maastricht University Maastricht the Netherlands; ^2^ Department of Mathematics and Statistics University of Calgary Calgary Canada; ^3^ Department of Economics, City Campus University of Antwerp Antwerp Belgium

**Keywords:** Bayesian optimal design, discrete choice experiment, interaction effects, model robust design, partial profile design, simulated annealing

## Abstract

Discrete Choice Experiments (DCEs) investigate participants' preferences by observing their choice behavior in hypothetical scenarios and are widely used in the domain of healthcare. To reduce participants' cognitive burden, especially when dealing with a large number of attributes, researchers often employ partial profile designs. In these designs, certain attributes within each choice set are kept constant. Current literature on partial profile designs mainly focuses on main‐effects models rather than interaction‐effect models, with certain partial profile designs even incapable of estimating interaction effects. To address this issue, this paper introduces a Simulated Annealing (SA) algorithm to construct partial profile designs based on an interaction‐effects model. During the experimental design phase, the existence and magnitude of interaction effects are often unknown. Therefore, this paper proposes a model‐robust experimental design strategy. Through extensive simulation experiments and a real‐life case study, we demonstrate that our SA model‐robust partial profile design performs relatively well regardless of the underlying model.

## 1 Introduction

Discrete choice experiments (DCEs) investigate individual preferences for attributes of products or services and have been widely applied in healthcare [1, 2]. In these experiments, participants are typically asked to select their preferred profile from a choice set composed of two or more profiles. These profiles are defined as combinations of levels of attributes. By analyzing the choice behavior of participants, researchers can gain insights into people's preferences for different attributes and levels, and make predictions about their decisions in real‐life scenarios.

Many DCEs in healthcare study a large number of attributes, including examples such as [3, 4, 5]. However, this abundance of attributes can result in an overload of information for respondents, significantly increasing their cognitive burden [6]. To simplify the choice task, Reference [7] suggested that in each choice set, respondents should be provided with profiles varying in only a limited set of attributes, while keeping the levels of the other attributes constant. These types of profiles are known as partial profiles, as opposed to full profiles, where all the attributes are varying in each choice set. Typically, the number of varying attributes in partial profiles is consistent across each choice set and is known as the profile strength [8]. Employing a partial profile design can effectively reduce response error arising from respondents' inattention, thereby achieving improved response efficiency compared to a full profile design [9, 10, 11]. For example, Reference [12] found in a randomized controlled DCE that using a partial profile design, compared to a full profile design, reduced the dropout rate by 30% and increased the level of choice consistency by 33%.

Due to the nonlinear nature of the choice model, the construction of optimal partial profile designs for DCEs necessitates knowledge of the model parameters' values. However, these parameter values are often unknown at the experimental design stage. To address this, three approaches have been proposed. The first approach, known as the utility‐neutral design, assumes that all unknown parameter values are zero. While straightforward, this method relies on an unrealistic assumption that respondents exhibit no distinct preference for different attribute levels. The second approach, locally optimal design, assigns predetermined nonzero values to the parameters [13]. Although more refined than the utility‐neutral design, this method remains sensitive to the accuracy of the specified parameter values. The third approach, the Bayesian optimal design, incorporates a prior distribution for the parameters in the choice model. By accounting for the uncertainties associated with the assumed parameter values, this method provides a better solution compared to a single specification of parameter values. Since its initial proposal by [14], the Bayesian design approach has found wide application in healthcare, for example, References [15, 16, 17]. In this study, we focus on the Bayesian design approach because it demonstrates superior performance compared to other design methods, particularly when there is an abundance of prior information [18].

Thus far, the majority of research on optimal partial profile designs in DCEs has concentrated on main‐effects models. Notable contributions to this field include [19, 20, 21, 22]. However, in the experimental design of DCEs, identifying and accurately estimating interaction effects are widely considered essential [23]. Although often overlooked by researchers, interaction effects, particularly two‐way interactions, frequently occur in practice and are important for understanding subjects' real‐life trade‐offs and competing preferences [24, 25, 26]. For example, Reference [9] highlighted that there is often a significant interaction effect between severity and duration of symptoms. Respondents are unable to evaluate the severity of a migraine without knowing its duration, and similarly, they cannot assess how long the migraine will last without understanding its severity within a specific period. Another healthcare intervention DCE, conducted by [27] and serving as the motivational example for this paper, revealed that the interaction terms between the type of healthcare intervention and the severity of the disease, as well as between the type of healthcare intervention and the patient's age, significantly influence the public's prioritization of healthcare interventions. Likelihood ratio tests indicated that the relative importance of these interaction terms was even higher than that of some main effects. Neglecting these interaction effects in a choice model may severely diminish its explanatory power [28].

To date, research on partial profile designs based on interaction‐effects models remains relatively scarce. Optimal designs are generally model‐specific, and designs optimized for main‐effects models may not perform well in the context of interaction‐effects models [29]. Some researchers have attempted to modify and adapt partial profile design algorithms developed for main‐effects models to make them suitable for interaction‐effects models. For instance, in [27], they made slight modifications to the algorithm of [21], retaining the structure of constant attributes from the main‐effects model while computing the optimal levels of these attributes specifically for the full interaction‐effects model. However, this strategy has three limitations. First, the structure of constant attributes determined by this method has significant room for improvement. One reason for this is that the algorithm of [20], when identifying constant attributes, focuses primarily on the number of levels for each attribute while overlooking any prior information associated with each attribute. Additionally, this algorithm is sequential in nature: in the first stage, it determines which attributes in each choice set are constant, and in the second stage, it selects the optimal levels for the attributes. This sequential structure often performs worse than algorithms based on simultaneous optimization in complex optimization tasks [30, 31, 32]. Second, in stage two, the algorithm of [20] employs a coordinate‐exchange (CE) algorithm [33, 34] on the master design obtained from stage one to determine the optimal levels of the varying attributes. However, the CE algorithm is likely to converge quickly to a local optimum because of its hill‐climbing nature, which can affect the quality of the final design obtained [35]. Third, when determining optimal attribute levels, they base their approach on a full interaction‐effects model. In practical applications, however, it is rare for all interactions to be statistically significant, and researchers more commonly retain only significant interactions in their models. As a result, an optimal design derived from a full interaction‐effects model may not perform well in the final model, which includes only significant interactions.

To the best of our knowledge, only [36, 37, 38] have discussed optimal partial profile designs for interaction‐effects models. However, these works have two limitations. First, their approach is applicable to specific cases where the choice set comprises only two profiles. Second, their methodology is based on the unrealistic assumption that respondents have equal preferences for all profiles, limiting its applicability to utility‐neutral designs, which generally provide less precise parameter estimates than Bayesian optimal designs.

To address these shortcomings, this paper introduces a Simulated Annealing (SA) algorithm based on simultaneous optimization for constructing Bayesian optimal partial‐profile interaction‐effects designs, without being constrained by the number of profiles or attributes. This SA algorithm can simultaneously optimize both constant and varying attributes, regardless of whether the model under study is main‐effects or interaction‐effects. Additionally, this SA algorithm accepts not only better solutions in each iteration but also accepts worse solutions with a certain probability, allowing it to explore a wider range of possible better partial profile designs. Through extensive simulation experiments, we demonstrate that our SA algorithm can generate better partial profile designs within the same time frame compared to the algorithm of [21].

In constructing interaction‐effects partial profile designs, a practical issue is that the existence and magnitude of interaction effects are often unknown. To mitigate the negative impact of model misspecifications on experimental design, we also applied our SA algorithm to the model‐robust designs proposed by [29]. Through comprehensive simulation experiments, we demonstrate that SA model‐robust partial profile designs consistently perform well under various model misspecifications. Additionally, we revisit the healthcare intervention DCE by [27] to show that our SA robust partial profile design achieves more precise parameter estimates in practical application compared to other experimental design approaches.

The remainder of this paper is organized as follows. In Section 2, we use a healthcare intervention DCE as an example to highlight the potential issues present in current partial profile designs based on interaction‐effects models. Section 3 introduces the multinomial logit (MNL) model for analyzing choice data and discusses how to construct a model‐robust Bayesian 𝒟‐optimal design based on this model. In Section 4, we propose an SA algorithm for the construction of optimal partial profile designs. In Section 5, we compare the performance of our integrated SA algorithm with the two‐stage CE algorithm by extensive simulation experiments. In Section 6, we demonstrate how to construct model‐robust partial profile designs and compare their estimation performance with that of main‐effects and interaction‐effects partial profile designs. In Section 7, we employ our SA algorithm to construct a model‐robust design, addressing common challenges encountered in the design of real life DCEs. Finally, Section 8 summarizes our findings and outlines potential directions for future research.

## 2 Motivating Example

Reference [27] conducted a DCE to investigate the public's distributive preferences regarding healthcare interventions. Their study involved seven attributes, as listed in Table A1 of Appendix A. Due to the large number of attributes being studied and to reduce participants' cognitive load, they considered a partial profile design where each choice set contains three constant attributes. Besides focusing on the main effects of the attributes, they were also interested in the two‐way interactions between $x_{1}$ and any of the other attributes. However, due to the exclusion of four unrealistic attribute combinations, among which $x_{1}=$ Curative & $x_{6}=$ After 20 years, and $x_{1}=$ Curative & $x_{6}=$ After 5 years, the interaction effects between $x_{1}$ and $x_{6}$ could not be estimated. In their work, the authors used effects‐type coding for all attributes. Consequently, they needed to estimate 15 main‐effects and 12 interaction‐effects parameters in total. To provide sufficient information content for the DCE, they constructed a Bayesian 𝒟‐optimal partial profile design involving 42 choice sets with 2 profiles in each choice set. The 42 choice sets were evenly distributed across three survey groups, each containing 14 choice sets. An example of a choice set appears in Figure A1 of Appendix A.

The complete details of the partial profile design are displayed in Table A2 of Appendix A, where the constant attributes in each choice set are highlighted in gray. This design is generated by the two‐stage algorithm proposed by [20]. In stage one, the algorithm determines which variables should be set as constants in each choice set using an attribute balance method inspired by balanced incomplete block designs (BIBDs) proposed by [39]. A BIBD arranges t levels of a single qualitative factor, termed treatments, into S blocks. Each block contains $t_{v}$ treatments, where $t_{v}<t$. In the context of partial profile design, a choice set can be viewed as a block, and the varying attributes within each choice set can be considered as treatments. BIBDs ensure precise parameter estimates of treatment effects $\alpha_{i}$ in a two‐way ANOVA model. This model can be expressed as

(1) $$Y_{\text{is}}=\alpha_{i}+\delta_{s}+\varepsilon_{\text{is}},$$

where $Y_{\text{is}}$ denotes the response of treatment i in block s, $\alpha_{i}$ is the average response of treatment i, $\delta_{s}$ represents the fixed block effects for block s, and $\varepsilon_{\text{is}}$ is the random error associated with the response of treatment i in block s. All random errors are assumed to be independently and normally distributed with a mean of zero and variance $\sigma^{2}$. In matrix notation, the two‐way ANOVA model can be expressed as

(2) Y=Qα+Zδ+ε,

where Y is a vector of dimension $r=S\times t_{v}$ representing the responses, Q is an r×t design matrix for the treatments, $\alpha ={\left(\alpha_{1},\ldots ,\alpha_{t}\right)}^{T}$ represents the mean responses for each treatment, Z is an r×(S−1) design matrix that maps treatments to blocks, $\delta ={\left(\delta_{1},\ldots ,\delta_{S-1}\right)}^{T}$ is the vector of block effects, and ε represents the random error vector.

BIBDs only exist for certain specified combinations of S, t, and $t_{v}$, which limits their applicability in practical settings. To address this, Reference [20] proposed constructing 𝒟‐optimal designs for the two‐way ANOVA model defined in Equation (2) by maximizing the determinant of the information matrix M for the parameter α and δ, given by:

(3) $$M=\left[\begin{array}{cc} Q^{T}Q & Q^{T}Z\\ Z^{T}Q & Z^{T}Z \end{array}\right].$$

In the master design obtained using the 𝒟‐optimality criterion, equal attention is paid to each attribute, which is why this method is called the attribute balance approach. However, in practice, attributes often have different numbers of levels. Attributes with more levels typically need to be assigned as varying attributes more frequently to achieve equally precise part‐worth estimates as those with fewer levels.

To address this, Reference [21] improved the algorithm by introducing two variance balance approaches that construct weighted 𝒜‐optimal designs for the ANOVA model defined in Equation (1). The weighted 𝒜‐optimal criterion is adopted instead of the 𝒟‐optimal criterion because it minimizes the weighted sum of the parameter variances, thereby directly targeting the balance of estimation precision across attributes with heterogeneous numbers of levels. In contrast, 𝒟‐optimal (attribute balance) designs treat all attributes equally and are only appropriate when all attributes have the same number of levels.

The weighted 𝒜‐optimal design seeks to minimize

(4) $${𝒜}_{w}=\sum_{i=1}^{t}w_{i}\mathrm{Var}\left({\hat{\alpha }}_{i}\right),$$

where $w_{i}$ is the weight assigned to the variance of the *i*th individual parameter estimator ${\hat{\alpha }}_{i}$ in the two‐way ANOVA model. The variances are given by the diagonal elements of the upper left‐hand submatrix of the inverse of the information matrix $M^{-1}$, which can be calculated as

(5) $${\left\{Q^{T}\left(I_{r}-Z{\left(Z^{T}Z\right)}^{-1}Z^{T}\right)Q\right\}}^{-1},$$

where $I_{r}$ is an r×r identity matrix.

In an ANOVA model, a larger $w_{i}$ indicates that the treatment effect $\alpha_{i}$ is expected to be estimated more precisely than other treatment effects. In a partial profile design, let $d_{i}$ represent the number of levels for the *i*th attribute. A larger $d_{i}$ suggests that this attribute requires more information for accurate estimation, and thus its corresponding weight should also be larger. Reference [21] introduces two methods to calculate these weights to construct an ${𝒜}_{w}$‐optimal master design, referring to these methods as variance balance I and variance balance II.

In the variance balance I approach, the weight $w_{i}$ is defined as the ratio of the number of part‐worth values for attribute i to the total number of part‐worth values:

(6) $$w_{i}=\frac{d_{i}-1}{\sum_{i=1}^{t}d_{i}-t}.$$

Variance balance I therefore increases the weight linearly with the number of levels, ensuring that the loss of information due to constant attributes is distributed evenly across all part‐worths.

In the variance balance II approach, the weight $w_{i}$ can be calculated as

(7) $$w_{i}=\frac{{\left(d_{i}-1\right)}^{2}}{2d_{i}}.$$

This formula is derived from the information matrix of utility‐neutral optimal full‐profile designs [19]. Compared with variance balance I, variance balance II enlarges the weight differences across attributes, giving proportionally greater weight to attributes with more levels or higher complexity. Hence, variance balance II is preferred when the goal is to achieve more precise estimation for attributes with many levels.

After obtaining the master design generated in stage one, References [20, 21] use a restricted CE algorithm to determine the optimal levels of the attributes. The CE algorithm initiates by creating a random initial design as a starting point. The algorithm iteratively optimizes this initial design by evaluating each attribute level across profiles. For each profile, the algorithm examines every attribute individually, calculating the optimality criterion for each level of that attribute. A level substitution is applied only if it results in an improved criterion value. This attribute‐by‐attribute examination continues across all profiles, completing one full cycle when the algorithm has identified the best possible exchange for each attribute in every profile. If any updates are made during this cycle, another complete iteration begins from the first attribute of the first profile. The process repeats until a full cycle is completed without any changes or until a specified maximum number of iterations is reached.

In partial profile design, References [20, 21] introduce an additional constraint to the CE algorithm's iterative process, specifically restricting the levels of the non‐constant attributes to vary within each choice set. This constraint prevents the occurrence of choice sets in the resulting optimal design where the number of varying attributes falls below $t_{v}$, characterizing it as the restricted CE algorithm.

Although the improved two‐stage algorithm accounts for heterogeneity in the number of levels of each attribute, it is not well developed for interaction‐effects models. This limitation arises because, in interaction‐effects models, even attributes with the same number of levels can be involved in different interactions. Reference [40] emphasized that the presence of interaction effects requires overlap in attribute levels within choice sets to generate the necessary contrasts for estimation. Therefore, if an attribute appears in multiple interaction terms within the model, it should remain constant more frequently to facilitate the estimation of these interaction effects. Consequently, considering only the differences in the number of levels among attributes is insufficient for accurately determining how fixed attributes should be distributed within choice sets. Moreover, the issue is further exacerbated by the fact that, in the two‐stage CE algorithm, the master design determined in stage one remains fixed in stage two. This constraint prevents any adjustments, even if the initially generated master design proves to be inefficient for estimating interaction effects within the model.

To address this, we propose a simultaneous‐optimization SA algorithm to handle this complex problem. Unlike the two‐stage algorithm, our SA algorithm uses the Bayesian ${𝒟}_{B}$‐optimality criterion to simultaneously optimize the allocation of constant attributes and the levels of all attributes, ensuring greater flexibility across different models under study. Moreover, as pointed out by [41], compared to the hill‐climbing CE algorithm, the SA algorithm is more likely to escape local optima and explore a broader experimental region, thus generating superior choice designs within a given time frame.

Beyond the algorithm, this healthcare DCE also encountered a common issue in the optimal design of interaction‐effects models: the misspecification of the underlying model. In the experimental design phase, they consider an MNL model with 12 interaction effects. Subsequent data analysis, however, indicated that only the interaction terms between $x_{1}$ and $x_{4}$ as well as $x_{1}$ and $x_{7}$ were significant. Consequently, they dropped the non‐significant interaction terms in the final model. It is important to note that optimal designs generated for a specific model do not necessarily perform well when applied to other models [42]. During the experimental design phase, the limited information about interactions complicates the accurate identification of the true underlying model. To address this, Reference [29] proposed a robust strategy for full profile designs that performs well even in the face of model misspecification. In this paper, we extend this strategy to partial profile designs and employ our SA algorithm to construct such model‐robust designs.

## 3 Methodology

### 3.1 Multinomial Logit Model Bayesian 𝒟‐Optimality

The MNL model is based on the assumption of utility maximization, which means that participants in the experiment will choose the profile with the highest utility in each choice set. Suppose each participant faces S different choice sets, with each choice set containing J distinct profiles. The perceived utility $U_{\mathrm{sj}}$ of a respondent choosing profile j in choice set s can be expressed as

(8) $$U_{\mathrm{sj}}=x_{\mathrm{sj}}^{T}\beta +\varepsilon_{\mathrm{sj}},$$

where $x_{\mathrm{sj}}$ represents the attribute vector, β is the vector of corresponding coefficients. The MNL model assumes that the random errors $\varepsilon_{\mathrm{sj}}$ are independently and identically distributed according to a Gumbel (or Type I extreme value) distribution. And therefore, the MNL probability $p_{\mathrm{sj}}$ that profile j is selected in choice set s can be expressed as

(9) $$p_{\mathrm{sj}}=\frac{\;\exp\left(x_{\mathrm{sj}}^{T}\beta \right)}{\sum_{j=1}^{J}\exp\left(x_{\mathrm{sj}}^{T}\beta \right)}.$$

The coefficient vector β can be estimated by maximizing the log‐likelihood function:

(10) $$\mathrm{LL}\left(\beta \right)=\sum_{n=1}^{N}\sum_{s=1}^{S}\sum_{j=1}^{J}y_{\mathrm{nsj}}\ln\left(p_{\mathrm{sj}}\right),$$

where $y_{\mathrm{nsj}}$ is a binary variable which equals one if respondent n chooses profile j from choice set s and zero otherwise. To construct optimal choice designs, many researchers employ the 𝒟‐optimality criterion [14], which leads to a 𝒟‐optimal design. The 𝒟‐optimal design obtains more precise parameter estimates by maximizing the determinant of the information matrix. The 𝒟‐optimality criterion is defined as

(11) 𝒟=log|M(X,β)|,

where X is the design matrix and M(X,β) is the information matrix of the model under study. In MNL models, the information matrix M(X,β) can be calculated as the sum of the information matrix of each choice set s, that is,

(12) $$M\left(X,\beta \right)=\sum_{s=1}^{S}X_{s}^{T}\left(P_{s}-p_{s}p_{s}^{T}\right)X_{s},$$

where $X=\left(X_{1},\ldots ,X_{S}\right)$ is the total model matrix for all S choice sets, $P_{s}=\mathrm{diag}\left(p_{s}\right)$ with $p_{s}={\left(p_{s1},\ldots ,p_{\mathrm{sJ}}\right)}^{T}$ records the MNL probabilities of all J profiles in choice set s.

According to Equation (9), the MNL probabilities are dependent on the parameter vector β, which is often unknown during the experimental design phase. To address this issue, researchers often assume that the parameter vector β follows a certain prior distribution, leading to a Bayesian optimal design approach [20]. Denote the prior distribution for β as π(β), the Bayesian 𝒟‐optimality criterion seeks to maximize the determinant of the information matrix averaged over π(β), that is,

(13) $${𝒟}_{B}=\int_{{ℛ}^{m}}\log\left|M\left(X,\beta \right)\right|\pi \left(\beta \right)\;d\;\beta .$$

Given that Equation (13) does not have a closed‐form solution, the ${𝒟}_{B}$‐optimality criterion is often numerically approximated through sampling from the prior distribution π(β). In our work, we utilize the spherical‐radial transformation sampling method proposed by [43], which has been shown to evaluate the Bayesian optimality criterion more efficiently than alternative approaches [44].

To compare the statistical efficiency of different partial profile designs, we use the relative ${𝒟}_{B}$‐efficiency as a measure [45]. The Bayesian ${𝒟}_{B}$‐efficiency of a design X relative to another design $X^{*}$ can be calculated as

(14) $${𝒟}_{B}‐\mathrm{eff}\left(X,X^{*}\right)=\exp\left(\frac{{𝒟}_{B}\left(X\right)-{𝒟}_{B}\left(X^{*}\right)}{m}\right),$$

where m is the dimension of the parameter vector β. A value of 1 indicates equal statistical efficiency for the two designs. A value greater than 1 indicates that the design X performs better and vice versa.

### 3.2 Model Robust Bayesian 𝒟‐Optimality

Due to the limited information about interaction effects during the experimental design phase, it is challenging to determine the underlying model. An optimal experimental design for a specific model may be inefficient in alternative models [42]. Consequently, we introduce a model‐robust experimental design strategy that ensures our design performs relatively well even when faced with model misspecification.

Let $\beta_{\text{main}}$ and $\beta_{\mathrm{int}}$ be the parameter vectors associated with these models, with dimensions $m_{\text{main}}$ and $m_{\mathrm{int}}$, respectively. According to the definition of the 𝒟‐optimality criterion in Equation (11), we have:

(15) $${𝒟}_{\text{main}}=\log\left|M\left(X,\beta_{\text{main}}\right)\right|,$$

and

(16) $${𝒟}_{\mathrm{int}}=\log\left|M\left(X,\beta_{\mathrm{int}}\right)\right|.$$

As in [29], our model‐robust design uses a composite design criterion based on both the main‐effects and interaction‐effects model, that is:

(17) $${𝒟}_{\text{robust}}=\frac{{𝒟}_{\text{main}}}{m_{\text{main}}}+\frac{{𝒟}_{\mathrm{int}}}{m_{\mathrm{int}}}.$$

Similarly, the Bayesian composite design criterion can also be calculated as the average over the parameter distribution.

## 4 Simulated Annealing Algorithm

Due to the nonlinear nature of the MNL model, constructing optimal choice designs often necessitates the use of a search algorithm. Currently, most algorithms used to construct Bayesian optimal experimental designs are based on hill‐climbing approaches, such as the CE algorithm [18, 33]. These algorithms, during iterations, accept only improved choice designs, thus leading to rapid convergence. To address this issue, Reference [46] has developed an SA algorithm for constructing Bayesian optimal designs for DCEs. Since the work of [46] focuses on full profile designs and could not be applied to partial profile designs, we have made several modifications based on their approach, resulting in the algorithm defined in Algorithm 1.

ALGORITHM 1 **Pseudo code for simulated annealing.**

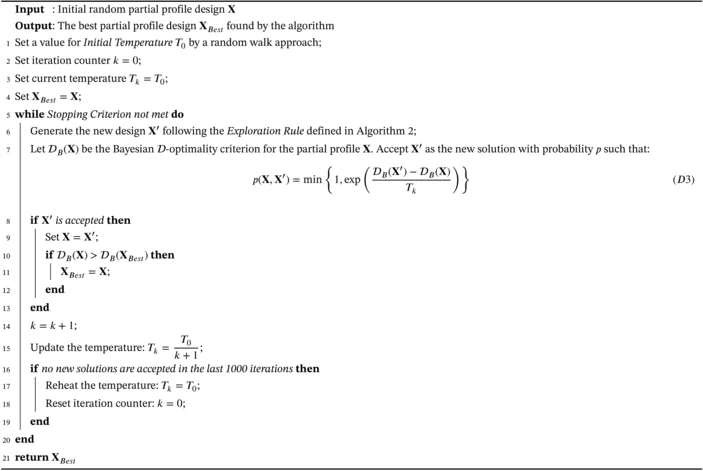

The SA algorithm is a probabilistic technique used to find or approximate the global optimum of a given objective function by simulating the process of slowly cooling a material to increase the stability of the system. This algorithm requires a random initial partial profile design X as input. It begins by generating a problem‐specific initial temperature $T_{0}$ through a random walk method as in [46]. Specifically, we perform a random walk of length 100 to estimate the maximum absolute difference ${\left|\Delta \right|}_{\max}$ in the Bayesian 𝒟‐optimality criterion between two consecutive designs. Following [46], we set the initial probability of accepting a worse solution to $p_{0}=0.99$. The initial temperature is then calculated as

(19) $$T_{0}=\frac{{\left|\Delta \right|}_{\max}}{|\log\left(p_{0}\right)|}.$$

Together with $T_{0}$, the iteration counter k is set to zero, and the initial design X is considered as the current best design $X_{\text{Best}}$. At the end of each iteration, the system temperature is updated using the hyperbolic cooling function $f\left(k,T_{0}\right)=\frac{T_{0}}{k+1}$. This cooling schedule decreases the temperature relatively quickly during the early iterations and more slowly during later iterations. Through extensive experiments, Reference [46] showed that this cooling function generated higher‐quality Bayesian optimal DCE designs than the geometric cooling function, although at the cost of a longer runtime. Simultaneously, a new random partial profile design $X^{\prime }$ is generated in the neighborhood of X based on the exploration rule as defined in Algorithm 2. Once $X^{\prime }$ is generated, its Bayesian 𝒟‐optimality criterion is calculated, and the Metropolis acceptance criterion [47], as defined in Equation (18), is used to decide whether to accept this new design. Unlike hill‐climbing algorithms, this algorithm accepts not only all superior solutions but also, with a certain probability, inferior ones. If the newly introduced solution is better than the current optimum, $X_{\text{Best}}$ is updated accordingly. As the algorithm progresses, the decrease in the temperature of the system gradually reduces the likelihood of accepting poorer solutions. At very low temperatures, the algorithm behaves similarly to a hill‐climbing approach, primarily accepting only superior solutions. To prevent premature convergence, if no new solutions are accepted within 1000 iterations, the system temperature is reheated to $T_{0}$, enabling exploration of more potential solutions. This process iterates continuously until the predetermined stopping criterion is met. Once the stopping criterion is satisfied, the algorithm terminates and outputs $X_{\text{Best}}$ as the optimal partial profile design. Typically, the stopping criterion can be defined as the algorithm running until either a predetermined maximum runtime is reached or the number of reheating cycles reaches a specified limit. Alternatively, a more adaptive criterion can be used, such as terminating the algorithm when no new best solution is found within an entire reheating cycle.

Compared to the work of [46], the primary distinction in our approach lies in the modification of the exploration rule. In their work, modifications to X were made by randomly altering the level of a randomly selected attribute within a randomly chosen choice set. However, this method does not address the constraint in constructing partial profile designs where each choice set must maintain a fixed number of attributes as constant.

ALGORITHM 2 **Pseudo code for exploration rule.**

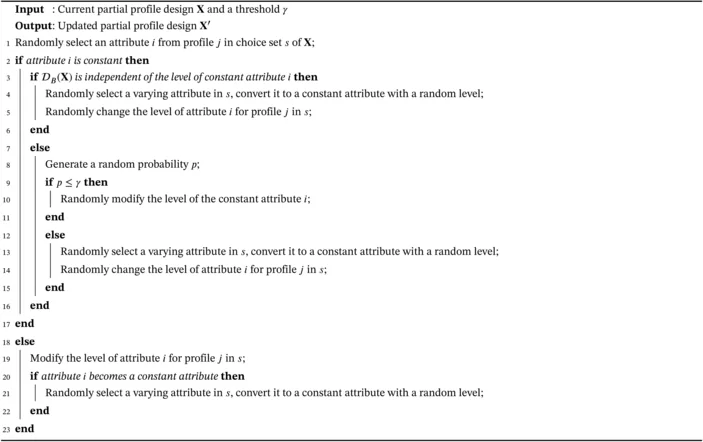

To address this limitation, we utilized the exploration rule defined in Algorithm 2. Our exploration rule begins by selecting a random attribute i from a randomly chosen profile j within a random choice set s of the current design X. We then check whether the attribute i is constant. If so, we assess whether changing the level of this constant attribute affects the Bayesian 𝒟‐optimality criterion, specifically to rule out:

1. Attribute i is not involved in any interaction effects that we aim to study.
2. All interaction effects involving this attribute in our model are comprised exclusively of other constant attributes from current choice set s.

If either of these conditions holds, changing the level of this constant attribute does not impact the information matrix. In such cases, we proceed by converting this constant attribute into a varying one and randomly selecting another varying attribute to be fixed at a random level, thereby maintaining the required structure of the partial profile design. Conversely, if modifying the attribute i affects the information matrix, we determine whether to alter its level based on a predetermined threshold γ. This threshold, ranging between 0 and 1, correlates directly with the probability of changing the level of a constant attribute. The higher the threshold, the greater the likelihood. In this study, we define γ as the ratio of constant attributes to the total number of attributes. If the selected attribute is not constant, we randomly modify its level. It is important to note that this operation may result in the attribute transforming from varying to constant. To maintain constant profile strength, under such circumstances, we convert a randomly selected constant attribute to varying. Our approach carefully accounts for the inherent constraints of partial profile designs while ensuring minimal perturbation to the existing design. This strategy balances structural constraints with sufficient exploration, allowing the algorithm to efficiently explore the design space during optimization.

## 5 Algorithm Comparison

In this section, we evaluate the performance of our SA algorithm and the algorithm of [21] through computational experiments. All code used in this study was developed in MATLAB R2024b and executed on a system powered by an 11th Gen Intel Core i5‐1135G7 processor.

### 5.1 Simulation Setup

To control the size of the experiment, we consider DCEs with 24 choice sets and 6 attributes, with attribute levels of 2, 2, 2, 3, 3, and 3, respectively. To obtain generalized conclusions, we consider various experimental settings defined by the number of profiles (J), the number of constant attributes (F), the number of two‐way interaction terms (Int), and the prior distributions.

For the number of profiles, we consider scenarios with 2 or 3 profiles. For the number of constant attributes, we consider scenarios with 1 or 2 constant attributes. Regarding the number of interaction terms, we consider four scenarios: no interaction terms, interactions between the first attribute and the other 2‐level attributes, interactions between the first attribute and all 3‐level attributes, and interactions between the first attribute and all other attributes. In our work, we employ effects‐type coding for all attributes, ensuring that the sum of the levels for each attribute equals zero, which necessitates the estimation of coefficients for all levels of each attribute excluding the last level. Correspondingly, the number of interaction terms is 0, 2, 6, and 8, respectively.

The prior distribution includes the prior mean $\beta_{0}$ and prior variance $\sum_{0}$. Regarding the prior mean $\beta_{0}^{\text{main}}$ for main effects, we assume that preferences for each attribute increase with the level, meaning that the first level is least favored and the last level is most favored. For generality, we define $\beta_{0}^{\text{main}}$ as a function of a scale parameter λ:

(20) $$\beta_{0}^{\text{main}}\left(\lambda \right)={\left(-\lambda ,-\lambda ,-\lambda ,-\lambda ,0,-\lambda ,0,-\lambda ,0\right)}^{T},$$

where $\lambda \in \left\{1,\frac{1}{2},\frac{1}{3}\right\}$. Similarly, we set the prior covariance matrix $\sum_{0}^{\text{main}}$ of the main effects as a function of a scale parameter κ:

(21) $$\sum_{0}^{\text{main}}\left(\kappa \right)=\left(\begin{array}{ccccccccc} \kappa^{2} & 0 & 0 & 0 & 0 & 0 & 0 & 0 & 0\\ 0 & \kappa^{2} & 0 & 0 & 0 & 0 & 0 & 0 & 0\\ 0 & 0 & \kappa^{2} & 0 & 0 & 0 & 0 & 0 & 0\\ 0 & 0 & 0 & \kappa^{2} & -0.5\kappa^{2} & 0 & 0 & 0 & 0\\ 0 & 0 & 0 & -0.5\kappa^{2} & \kappa^{2} & 0 & 0 & 0 & 0\\ 0 & 0 & 0 & 0 & 0 & \kappa^{2} & -0.5\kappa^{2} & 0 & 0\\ 0 & 0 & 0 & 0 & 0 & -0.5\kappa^{2} & \kappa^{2} & 0 & 0\\ 0 & 0 & 0 & 0 & 0 & 0 & 0 & \kappa^{2} & -0.5\kappa^{2}\\ 0 & 0 & 0 & 0 & 0 & 0 & 0 & -0.5\kappa^{2} & \kappa^{2} \end{array}\right),$$

where $\kappa \in \left\{1,\frac{1}{2},\frac{1}{3}\right\}$. For attributes with three levels, we ensure a negative correlation between the parameters of the first two levels, thus maintaining equal variances across all parameters, particularly for the third level [48]. By combining different values of λ and κ, we obtain a total of nine distinct prior distributions for the main effect parameters, broadly covering most common scenarios encountered in practice.

Regarding the prior distribution for interaction effects, we apply a naive prior, setting the prior mean to zero and the variance to one. This choice corresponds with the typically limited information available on interaction effects in most practical scenarios. For further discussion on the strategies of setting prior information for interaction effects, please refer to Section 6.

Utilizing distinct levels of these five key parameters that define the DCE settings, we implemented a full factorial experiment encompassing 2×2×4×3×3=144 different DCE scenarios. In each scenario, we constructed Bayesian optimal experimental designs using both the two‐stage CE algorithm of [21] and our SA algorithm.

To ensure a fair comparison, we determined the computational budget following the approach used in [41]. Specifically, for each scenario, we ran the CE algorithm from 30 random starting designs and recorded their combined runtime. The SA algorithm was then allowed to run for the same total amount of time, so that the two algorithms were compared under an equal overall computational budget.

Regarding the CE algorithm's parameter settings, in stage one, we apply the variance balance II approach, as introduced in Equation (7), to generate the master design. In stage two, we initialize the algorithm with 30 random starting points. To guarantee full convergence of the CE algorithm, we terminate it at each starting point when its ${𝒟}_{B}$‐value shows no improvement over an entire cycle. Finally, among the 30 starting points, the best partial profile design obtained is selected as the final output of the CE algorithm.

### 5.2 Results

In each experimental setting, we documented the final partial profile designs $X_{\mathrm{CE}}$ and $X_{\mathrm{SA}}$ generated by these two algorithms and calculated the relative ${𝒟}_{B}$‐efficiency of the CE designs compared to the SA designs labeled as ${𝒟}_{B}-\mathrm{eff}$, which are detailed in Table 1. The results in the table indicate that the ${𝒟}_{B}‐\mathrm{eff}$ is less than 1 in all 144 scenarios, demonstrating the superior performance of the SA designs. On average, the relative ${𝒟}_{B}$‐efficiency across all 144 scenarios is about 92.60%. We further conducted a Wilcoxon signed‐rank test and found that the median of ${𝒟}_{B}‐\mathrm{eff}$ was significantly less than 1, with a *p*‐value less than 0.0001.

**TABLE 1:** Relative ${𝒟}_{B}$‐efficiency of the CE designs compared to the SA designs based on different experimental settings.

J	F	Int	λ	κ	${𝒟}_{B}‐\mathrm{eff}$	Runtime (s)	J	F	Int	λ	κ	${𝒟}_{B}‐\mathrm{eff}$	Runtime (s)
2	1	0	1	1	94.26%	534.96	3	1	0	1	1	92.97%	599.64
2	1	0	1	0.5	95.51%	431.69	3	1	0	1	0.5	96.28%	580.90
2	1	0	1	0.33	95.57%	419.73	3	1	0	1	0.33	95.98%	598.21
2	1	0	0.5	1	94.77%	489.28	3	1	0	0.5	1	94.57%	588.42
2	1	0	0.5	0.5	97.76%	430.61	3	1	0	0.5	0.5	95.38%	585.25
2	1	0	0.5	0.33	96.13%	409.03	3	1	0	0.5	0.33	93.97%	561.61
2	1	0	0.33	1	94.71%	515.55	3	1	0	0.33	1	95.69%	625.68
2	1	0	0.33	0.5	96.14%	443.94	3	1	0	0.33	0.5	95.92%	547.13
2	1	0	0.33	0.33	96.97%	420.64	3	1	0	0.33	0.33	95.28%	535.33
2	1	2	1	1	91.35%	774.73	3	1	2	1	1	89.02%	998.45
2	1	2	1	0.5	96.35%	745.11	3	1	2	1	0.5	94.61%	813.16
2	1	2	1	0.33	97.77%	636.07	3	1	2	1	0.33	95.17%	868.89
2	1	2	0.5	1	89.23%	813.96	3	1	2	0.5	1	91.44%	990.33
2	1	2	0.5	0.5	97.37%	773.28	3	1	2	0.5	0.5	94.80%	957.95
2	1	2	0.5	0.33	98.22%	689.98	3	1	2	0.5	0.33	95.46%	936.71
2	1	2	0.33	1	90.22%	923.66	3	1	2	0.33	1	89.38%	961.40
2	1	2	0.33	0.5	96.83%	776.80	3	1	2	0.33	0.5	95.30%	855.32
2	1	2	0.33	0.33	99.21%	731.94	3	1	2	0.33	0.33	95.08%	951.75
2	1	6	1	1	86.14%	2578.77	3	1	6	1	1	86.54%	2085.88
2	1	6	1	0.5	94.64%	2011.19	3	1	6	1	0.5	90.77%	1681.61
2	1	6	1	0.33	95.20%	2294.73	3	1	6	1	0.33	91.78%	1573.64
2	1	6	0.5	1	89.93%	1712.03	3	1	6	0.5	1	85.52%	2134.28
2	1	6	0.5	0.5	94.05%	1364.89	3	1	6	0.5	0.5	93.26%	1859.44
2	1	6	0.5	0.33	96.45%	1458.95	3	1	6	0.5	0.33	94.84%	2085.58
2	1	6	0.33	1	86.52%	1822.05	3	1	6	0.33	1	87.43%	2128.47
2	1	6	0.33	0.5	93.97%	1365.48	3	1	6	0.33	0.5	93.65%	1714.35
2	1	6	0.33	0.33	96.37%	1432.95	3	1	6	0.33	0.33	94.84%	1490.76
2	1	8	1	1	87.37%	3700.49	3	1	8	1	1	83.62%	3301.11
2	1	8	1	0.5	94.95%	2604.04	3	1	8	1	0.5	91.21%	2896.04
2	1	8	1	0.33	93.18%	2812.72	3	1	8	1	0.33	91.41%	2698.85
2	1	8	0.5	1	86.91%	3666.94	3	1	8	0.5	1	83.00%	3237.76
2	1	8	0.5	0.5	97.60%	2760.64	3	1	8	0.5	0.5	91.36%	2759.62
2	1	8	0.5	0.33	98.77%	2621.92	3	1	8	0.5	0.33	93.66%	2830.41
2	1	8	0.33	1	86.45%	3704.42	3	1	8	0.33	1	82.80%	3783.41
2	1	8	0.33	0.5	97.01%	2338.69	3	1	8	0.33	0.5	92.68%	2958.64
2	1	8	0.33	0.33	97.42%	2678.60	3	1	8	0.33	0.33	91.95%	2655.95
2	2	0	1	1	92.99%	485.43	3	2	0	1	1	94.61%	545.25
2	2	0	1	0.5	93.45%	448.19	3	2	0	1	0.5	95.00%	564.94
2	2	0	1	0.33	94.71%	412.52	3	2	0	1	0.33	94.31%	577.94
2	2	0	0.5	1	91.11%	449.15	3	2	0	0.5	1	95.45%	649.61
2	2	0	0.5	0.5	93.87%	426.69	3	2	0	0.5	0.5	97.14%	532.09
2	2	0	0.5	0.33	95.50%	389.20	3	2	0	0.5	0.33	94.11%	572.81
2	2	0	0.33	1	91.87%	441.52	3	2	0	0.33	1	95.99%	585.26
2	2	0	0.33	0.5	95.03%	432.88	3	2	0	0.33	0.5	96.41%	519.03
2	2	0	0.33	0.33	95.74%	353.91	3	2	0	0.33	0.33	96.68%	535.36
2	2	2	1	1	90.59%	772.53	3	2	2	1	1	90.59%	882.89
2	2	2	1	0.5	93.20%	648.75	3	2	2	1	0.5	92.24%	923.63
2	2	2	1	0.33	92.58%	634.11	3	2	2	1	0.33	91.25%	853.95
2	2	2	0.5	1	88.43%	775.68	3	2	2	0.5	1	93.61%	889.52
2	2	2	0.5	0.5	94.40%	677.47	3	2	2	0.5	0.5	94.21%	1173.93
2	2	2	0.5	0.33	94.79%	707.98	3	2	2	0.5	0.33	92.16%	1081.97
2	2	2	0.33	1	87.43%	715.98	3	2	2	0.33	1	93.13%	1100.29
2	2	2	0.33	0.5	94.09%	650.83	3	2	2	0.33	0.5	95.76%	919.08
2	2	2	0.33	0.33	93.18%	602.58	3	2	2	0.33	0.33	92.40%	787.88
2	2	6	1	1	85.26%	2106.61	3	2	6	1	1	87.81%	1673.69
2	2	6	1	0.5	90.67%	2081.86	3	2	6	1	0.5	91.83%	2500.54
2	2	6	1	0.33	93.43%	2286.87	3	2	6	1	0.33	93.82%	1517.69
2	2	6	0.5	1	85.20%	1992.59	3	2	6	0.5	1	89.60%	1801.03
2	2	6	0.5	0.5	93.06%	1706.00	3	2	6	0.5	0.5	92.77%	1627.68
2	2	6	0.5	0.33	93.67%	2178.80	3	2	6	0.5	0.33	93.05%	1571.33
2	2	6	0.33	1	82.58%	2804.54	3	2	6	0.33	1	88.30%	1723.68
2	2	6	0.33	0.5	93.10%	2286.08	3	2	6	0.33	0.5	94.98%	1412.99
2	2	6	0.33	0.33	94.23%	2140.95	3	2	6	0.33	0.33	92.95%	1567.71
2	2	8	1	1	87.29%	3582.03	3	2	8	1	1	84.77%	2696.18
2	2	8	1	0.5	90.56%	3347.02	3	2	8	1	0.5	90.49%	3469.51
2	2	8	1	0.33	91.34%	3235.38	3	2	8	1	0.33	89.59%	3762.90
2	2	8	0.5	1	89.02%	3682.26	3	2	8	0.5	1	86.96%	2995.31
2	2	8	0.5	0.5	92.34%	3034.45	3	2	8	0.5	0.5	90.24%	2699.46
2	2	8	0.5	0.33	93.06%	3116.88	3	2	8	0.5	0.33	90.86%	2563.88
2	2	8	0.33	1	89.28%	4602.25	3	2	8	0.33	1	85.99%	2809.23
2	2	8	0.33	0.5	93.11%	2949.69	3	2	8	0.33	0.5	93.79%	2718.60
2	2	8	0.33	0.33	92.27%	3249.27	3	2	8	0.33	0.33	91.76%	2335.32

Among the five factors that define the DCE scenarios, two have a particularly strong impact on the relative performance of the algorithms: the size of the prior variance κ and the number of two‐way interaction terms Int. Regarding κ, our findings align with those of [46], showing that the advantage of the SA algorithm tends to be more pronounced as κ increases. This is because larger prior variances indicate greater uncertainty in the prior information, making the optimization objective function more complex and increasing the likelihood of encountering multiple local optima. In such cases, the SA algorithm's ability to accept inferior solutions during the search process allows it to escape local optima more effectively, explore a broader design space, and ultimately identify superior designs.

As for Int, the relative performance of the SA algorithm generally improves as the number of interaction terms in the model increases. This is primarily because the first stage of the two‐stage CE algorithm does not account for interaction effects. As the influence of interaction effects in the model grows, this omission becomes more consequential, leading to a greater discrepancy between the initially generated master design and the structure of the optimal design. Consequently, the integrated SA algorithm, which does not rely on a predetermined master design, is better suited to adapt to the complexities introduced by interaction effects, thereby achieving superior performance.

Two additional computational analyses complement the main algorithm comparison. Appendix C provides a paired run‐time benchmark that directly compares the convergence behavior of SA and CE from common starting designs. Across 120 paired runs, SA reached the final criterion value obtained by CE in every case and typically did so in only 14%–34% of the time required for CE to converge. Appendix D examines the scalability of SA by separating the number of iterations required for convergence from the computational cost per iteration as the number of profiles and attributes increases. Together, these analyses provide further evidence on the computational efficiency and practical scalability of the proposed SA algorithm.

## 6 Model Robust Partial Profile Design

In this section, we demonstrate how to use our SA algorithm to construct a model‐robust Bayesian 𝒟‐optimal partial profile design and compare its structure with those of the main‐effects and interaction‐effects designs. Subsequently, we evaluate the stability of these three design strategies under various misspecifications of the model and prior information by evaluating their performance through relative ${𝒟}_{B}$‐efficiency.

### 6.1 Design Construction

We consider a DCE consisting of 24 choice sets, each containing two profiles. The experiment includes six attributes, each with levels distributed as 2, 2, 2, 3, 3, and 3. In each choice set, one attribute remains constant across all profiles. Effects‐type coding is again employed for all attributes. Consequently, the main‐effects parameter dimension $m_{\text{main}}=2+2+2+3+3+3-6=9$. In the interaction‐effects model, in addition to the main effects, we incorporate two‐way interactions between the first attribute and the second and fourth attributes, thereby increasing the parameter dimension $m_{\mathrm{int}}$ to 12.

Regarding the prior distribution of the main‐effects model, we set the prior mean $\beta_{0}^{\text{main}}$ as

$$\beta_{0}^{\text{main}}={\left(-1,-1,-1,-1,0,-1,0,-1,0\right)}^{T},$$

and the prior covariance matrix $\sum_{0}^{\text{main}}$ as

$$\sum_{0}^{\text{main}}=\left(\begin{array}{ccccccccc} 1 & 0 & 0 & 0 & 0 & 0 & 0 & 0 & 0\\ 0 & 1 & 0 & 0 & 0 & 0 & 0 & 0 & 0\\ 0 & 0 & 1 & 0 & 0 & 0 & 0 & 0 & 0\\ 0 & 0 & 0 & 1 & -0.5 & 0 & 0 & 0 & 0\\ 0 & 0 & 0 & -0.5 & 1 & 0 & 0 & 0 & 0\\ 0 & 0 & 0 & 0 & 0 & 1 & -0.5 & 0 & 0\\ 0 & 0 & 0 & 0 & 0 & -0.5 & 1 & 0 & 0\\ 0 & 0 & 0 & 0 & 0 & 0 & 0 & 1 & -0.5\\ 0 & 0 & 0 & 0 & 0 & 0 & 0 & -0.5 & 1 \end{array}\right).$$

For interaction effects, we again employed a naive prior, setting the prior mean to zero and the variance to one. That is, $\beta_{0}^{\mathrm{int}}=\left(\beta_{0}^{\text{main}},0_{3}\right)$ and $\sum_{0}^{\mathrm{int}}=\left[\begin{array}{cc} \sum_{0}^{\text{main}} & \\ & I_{3} \end{array}\right]$. Based on the prior information discussed above, we applied the three different Bayesian 𝒟–optimality criteria outlined in Equations ((15), (16), (17)) as the objective functions for the SA algorithm to generate the corresponding optimal partial profile designs. For each design, the SA algorithm employed an adaptive stopping criterion, which terminated the optimization process when no improvement in the ${𝒟}_{B}$ value was observed over an entire reheating cycle.

Table 2 presents the three partial profile designs obtained using different Bayesian 𝒟‐optimality criteria, where the constant attribute is highlighted in gray.

**TABLE 2:** Comparison of partial profile designs using three different strategies for DCEs with 24 choice sets, each containing 2 profiles and 1 constant attribute.

	Main‐effects						Interaction‐effects						Robust					
Choice set	1	2	3	4	5	6	1	2	3	4	5	6	1	2	3	4	5	6
1	2	1	1	2	3	3	1	2	1	2	2	3	2	2	1	2	1	1
1	2	2	2	3	1	1	1	1	2	3	3	1	1	1	1	1	3	3
2	1	2	1	1	2	2	2	1	1	3	1	3	2	2	1	3	1	3
2	1	1	2	2	1	1	2	2	2	1	2	2	1	2	2	2	3	2
3	1	2	2	2	1	2	2	1	2	3	3	3	2	1	2	3	2	1
3	2	2	1	1	2	3	1	2	2	2	2	2	2	2	1	2	1	3
4	1	2	2	2	1	3	1	2	2	3	1	2	2	2	2	1	1	1
4	2	1	2	1	2	2	2	1	2	1	2	1	2	1	1	3	2	2
5	2	1	2	1	2	2	2	2	1	2	1	3	1	2	2	1	1	2
5	1	1	1	3	3	1	2	1	2	3	2	1	1	1	1	3	2	3
6	1	1	1	3	3	2	2	2	1	2	3	1	1	1	1	3	3	2
6	1	2	2	1	1	3	2	1	2	3	1	2	2	1	2	2	1	3
7	1	2	1	1	2	3	2	2	1	1	1	2	1	2	1	1	2	1
7	1	1	2	3	3	2	1	2	2	2	2	1	2	1	1	2	3	2
8	1	1	2	1	1	3	2	1	2	2	1	3	2	1	1	2	3	2
8	2	1	1	2	2	2	2	2	1	1	3	1	2	2	2	1	2	3
9	1	1	1	3	1	3	1	1	2	1	3	2	1	2	1	2	1	1
9	1	2	2	1	2	1	1	2	1	3	1	1	2	1	1	1	2	2
10	1	2	1	2	3	1	1	1	2	2	2	3	1	1	2	1	3	2
10	1	1	2	3	1	3	1	2	1	1	1	2	1	2	1	2	2	3
11	2	1	2	1	2	1	2	2	1	3	1	2	2	2	1	2	3	1
11	2	2	1	2	1	2	1	2	2	1	3	3	2	1	2	1	1	3
12	1	2	2	3	3	2	2	2	2	2	1	1	1	2	2	2	1	2
12	2	1	2	2	2	1	1	2	1	3	3	2	2	2	1	1	3	1
13	1	2	2	2	2	1	1	2	2	3	1	1	1	2	2	1	3	3
13	2	2	1	1	1	2	1	1	1	1	3	3	2	2	1	3	1	2
14	2	2	1	3	1	1	1	1	2	1	3	1	1	1	2	3	1	3
14	1	2	2	1	3	3	2	2	1	1	1	3	2	1	1	1	2	1
15	1	2	2	2	2	1	1	2	2	2	1	2	1	2	2	3	1	1
15	2	1	2	1	3	3	1	1	1	3	2	3	1	1	1	2	3	3
16	1	1	1	2	3	2	1	2	2	1	1	3	1	2	1	1	3	1
16	2	2	1	3	1	1	2	1	2	2	2	1	1	1	2	3	2	2
17	2	1	2	3	2	2	2	2	2	1	1	1	1	2	1	3	1	3
17	2	2	1	1	3	3	2	1	1	2	3	2	2	2	2	1	2	1
18	1	2	2	1	3	2	1	1	2	2	1	1	1	2	1	1	2	2
18	2	1	2	2	2	3	1	2	1	1	2	2	1	1	2	2	1	1
19	2	2	1	3	3	1	1	1	2	3	2	2	2	1	2	2	1	2
19	2	1	2	1	1	2	2	2	1	3	3	1	1	2	2	3	3	1
20	2	1	2	1	3	1	1	2	2	1	1	1	1	1	2	1	2	3
20	1	2	2	2	2	2	1	1	1	2	3	2	2	1	1	3	3	1
21	1	2	1	1	1	2	1	1	2	2	1	2	2	2	1	3	3	2
21	2	1	1	2	3	1	2	1	1	1	2	1	2	1	2	2	2	1
22	1	2	2	1	3	2	1	1	2	1	2	3	2	1	1	1	1	3
22	2	2	1	2	1	3	1	2	1	2	3	1	1	1	2	2	2	1
23	2	2	2	1	1	1	2	2	1	3	2	2	1	1	2	3	1	1
23	1	1	2	3	2	3	2	1	2	1	1	3	1	2	1	2	2	2
24	2	2	2	2	1	2	2	2	1	3	1	1	2	1	2	2	2	1
24	1	2	1	3	2	3	2	1	2	1	3	2	2	2	1	1	1	2

In the main‐effects design, the constant attribute is almost evenly distributed among the three 2‐level attributes, with no overlap observed among the 3‐level attributes. This finding aligns with [22], as when only main effects are considered, the 3‐level attributes require more parameters to be estimated. Consequently, they need to vary more frequently to ensure accurate estimation of their parameter values.

In the interaction‐effects design, the first attribute is fixed significantly more often, appearing as the constant attribute in 15 choice sets. Additionally, the 3‐level attribute 4 is fixed in 2 choice sets. This observation is consistent with the findings of [29], which indicate that attributes involved in interaction terms are more likely to exhibit overlap to provide the necessary contrasts for estimating interaction effects [40].

The robust design exhibits a structure that falls between the two. On one hand, similar to the main‐effects design, attribute overlap occurs only among the 2‐level attributes. On the other hand, as in the interaction‐effects model, the first attribute, which is involved in multiple interaction terms, is fixed more frequently. Correspondingly, the third attribute, which does not participate in interaction effects, is fixed less often than in the main‐effects design.

### 6.2 Sensitivity to Model and Prior Misspecifications

We compare the performance of the three designs presented in Table 2 under the following four true model specifications:

1. Model I: No interaction terms are present, meaning the true prior mean is given by
$$\beta_{0}^{\text{true}}={\left(-1,-1,-1,-1,0,-1,0,-1,0\right)}^{T}.$$

2 Model II: Only the interaction between Attribute 1 and Attribute 2 is present, with the true prior mean given by
$$\beta_{0}^{\text{true}}={\left(-1,-1,-1,-1,0,-1,0,-1,0,\lambda \right)}^{T}.$$

3 Model III: Only the interaction between Attribute 1 and Attribute 4 is present, with the true prior mean given by
$$\beta_{0}^{\text{true}}={\left(-1,-1,-1,-1,0,-1,0,-1,0,\lambda ,\lambda \right)}^{T}.$$

4 Model IV: Interactions between Attribute 1 and both Attributes 2 and 4 are present, with the true prior mean given by
$$\beta_{0}^{\text{true}}={\left(-1,-1,-1,-1,0,-1,0,-1,0,\lambda ,\lambda ,\lambda \right)}^{T}.$$

In this setup, λ acts as a scale factor for the interaction effects, with λ∈{0.1,0.2,0.3}. For each true model specification, we vary the value of λ, resulting in a total of 10 different true prior means.

Regarding the true prior variance, the prior covariance matrix for the main effects is set as $\sum_{0}^{\text{main}}$. For the interaction effects, we assume a prior variance of zero to precisely assess the impact of the interaction size on the design efficiency.

Using each true prior distribution, we generate a benchmark partial profile design that serves as a reference under the corresponding prior specification. For each case, we compare the ${𝒟}_{B}$‐efficiency of the three designs from Table 2 relative to the corresponding benchmark design. Notably, under Model I, the benchmark design is set as the main‐effects design, implying that in this scenario, the relative ${𝒟}_{B}$‐efficiency of the main‐effects design is equal to 1.

Table 3 presents the relative ${𝒟}_{B}$‐efficiency of the three designs compared to the benchmark design across 10 different scenarios. The results show that the main‐effects design performs worse when interaction effects are present in the underlying model. Specifically, as the number and magnitude of interaction effects increase, the efficiency of the main‐effects design declines further. Conversely, the interaction‐effects design exhibits the worst relative performance when the number of interaction terms is small. However, as the number of interaction terms increases, its relative performance improves.

**TABLE 3:** Comparison of ${𝒟}_{B}$‐efficiency of the three design strategies relative to the benchmark design in different DCE settings.

Model	λ	Main‐effect	Interaction‐effect	Robust
I	—	100.00%	87.23%	97.88%
II	0.1	90.94%	89.66%	97.79%
II	0.2	90.85%	89.59%	97.46%
II	0.3	90.14%	88.96%	96.42%
III	0.1	82.83%	93.24%	97.04%
III	0.2	81.57%	91.94%	96.25%
III	0.3	80.41%	90.84%	95.63%
IV	0.1	76.01%	92.32%	95.03%
IV	0.2	75.56%	91.63%	95.02%
IV	0.3	74.33%	89.90%	93.93%
Average		84.26%	90.53%	96.25%

The robust design consistently demonstrates strong performance across all considered scenarios, with an average relative ${𝒟}_{B}$‐efficiency of 96.25%. Notably, when interaction effects are present, its relative ${𝒟}_{B}$‐efficiency is the highest among the three designs in every case.

These findings highlight that the robust design is significantly more stable against model misspecification than the main‐effects and interaction‐effects designs. When prior information is limited and the underlying model is uncertain, employing the model‐robust Bayesian 𝒟‐optimality criterion for constructing partial profile designs proves to be a more reliable approach.

## 7 Healthcare Intervention Case Study

In this section, we revisit the healthcare intervention DCE conducted by [27], identifying potential experimental design issues outlined in Section 2. To address these concerns, we propose several alternative partial profile designs and compare their Bayesian 𝒟‐efficiency. Subsequently, we conduct simulation experiments to evaluate the accuracy of parameter estimation across these designs.

### 7.1 Case Study Setup

At the experimental design stage, Reference [27] constructed a Bayesian 𝒟‐optimal design based on an interaction‐effects MNL model which includes 27 parameters. The prior mean for the main effects was specified as 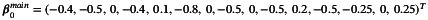. For the prior variance–covariance matrix, they let $\sum_{0}^{\text{main}}=\left[\begin{array}{ccccccc} A_{1} & & & & & & \\ & A_{2} & & & & & \\ & & A_{2} & & & & \\ & & & A_{2} & & & \\ & & & & A_{2} & & \\ & & & & & A_{2} & \\ & & & & & & A_{3} \end{array}\right]$, where $A_{1}=\left[0.09\right]$, $A_{2}=\left[\begin{array}{cc} 0.09 & -0.045\\ -\;0.045 & 0.09 \end{array}\right]$, and $A_{3}=\left[\begin{array}{cccc} 0.09 & -0.0225 & -0.0225 & -0.0225\\ -\;0.0225 & 0.09 & -0.0225 & -0.0225\\ -\;0.0225 & -0.0225 & 0.09 & -0.0225\\ -\;0.0225 & -0.0225 & -0.0225 & 0.09 \end{array}\right]$. Regarding the interaction effects, they assumed both the prior mean and variance to be 0. Using this prior information, they constructed a Bayesian optimal design for the 42 choice sets.

After combining the data from the three survey groups, they initially analyzed it using the same 27‐parameter MNL model employed during the experimental design stage. Using likelihood ratio tests, they found that only the interaction terms between $x_{1}$ and $x_{4}$ as well as $x_{1}$ and $x_{7}$ were statistically significant. After dropping the non‐significant interaction terms, they obtained a final model with the number of parameters reduced from 27 to 21 compared to the model used in the experimental design. Our case study evaluates the performance of different partial profile designs when lacking prior information for the interaction effects.

### 7.2 Alternative Partial Profile Designs

We consider four different Bayesian 𝒟‐optimal partial profile designs:

1. Main‐effects design: This is a Bayesian partial profile design that focuses solely on the main effects of the attributes. To calculate the Bayesian 𝒟‐optimality criterion, we used the prior distribution $N\left(\beta_{0}^{\text{main}},\sum_{0}^{\text{main}}\right)$, where the specific values of $\beta_{0}^{\text{main}}$ and $\sum_{0}^{\text{main}}$ are provided earlier in Section 7.1.
2. Original design: This is the original design employed in [27], which is detailed in Table A2.
3. Robust design: This is a Bayesian partial profile design that seeks to maximize the composite design criterion defined in Equation (17). For the main‐effects part, we also used the same prior parameters $\beta_{0}^{\text{main}}$ and $\sum_{0}^{\text{main}}$. As for the interaction‐effects part, we also consider all the two‐way interactions of interest and apply a naive prior distribution, where the prior mean is set to zero, and the variance is set to one. That is, $\beta_{0}^{\mathrm{int}}=\left(\beta_{0}^{\text{main}},0_{12}\right)$ and $\sum_{0}^{\mathrm{int}}=\left[\begin{array}{cc} \sum_{0}^{\text{main}} & \\ & I_{12} \end{array}\right]$.
4. True model design: A benchmark design was generated based on the final model obtained after the likelihood ratio tests, incorporating all main effects and only the interactions between $x_{1}$ and $x_{4}$ as well as $x_{1}$ and $x_{7}$, representing an ideal scenario where information on interaction effects is fully available. For consistency, we used the same prior $\beta_{0}^{\text{main}}$ and $\sum_{0}^{\text{main}}$ for main effects. Regarding interaction effects, we used the results of the data analysis from the study of [27] as prior information. The specific values are available in Table 4. Combining the two, we obtained the prior distribution for the true model, denoted as $N\left(\beta_{0}^{\text{true}},\sum_{0}^{\text{true}}\right)$.

**TABLE 4:** Prior information of the interaction effects in the true model.

	$x_{11}x_{41}$	$x_{11}x_{42}$	$x_{11}x_{71}$	$x_{11}x_{72}$	$x_{11}x_{73}$	$x_{11}x_{74}$
Mean	−0.0431	0.0345	0.012	−0.0676	−0.048	0.1103
SD	0.0378	0.0394	0.0528	0.0524	0.0558	0.0578

Using the prior information mentioned above, we employed our SA algorithm to construct the main‐effects design, robust design, and true model design. Similar to the work of [27], we also excluded unrealistic attribute level combinations. The specific details of these designs are detailed in Table B1 in Appendix B.

Table 5 records the Bayesian ${𝒟}_{B}$‐efficiency of each design relative to the true model design. The main‐effects design performed the worst, with an efficiency of only 75.89% relative to the true model design. This emphasizes the importance of considering interaction effects during the experimental design stage. The original design also performed poorly, only slightly better than the main‐effects design. This is partly due to the limitations of the design algorithm and partly to the inclusion of an excessive number of interaction terms not present in the true model. In contrast, the robust design performed better, achieving a ${𝒟}_{B}$‐efficiency of nearly 90% relative to the true model design.

**TABLE 5:** ${𝒟}_{B}$‐efficiency of four different designs relative to the true model design.

	Main‐effects	Original	Robust	True model
${𝒟}_{B}$‐efficiency	75.89%	78.64%	89.70%	100%

### 7.3 Comparison of Parameter Estimate Accuracy

We evaluated the estimation accuracy of different designs through simulation experiments. Specifically, we assumed a total of 300 participants in the DCE, evenly distributed across three survey groups. For each partial profile design, we simulated responses under the assumption that $\beta_{0}^{\text{true}}$ represents the true parameter vector, denoted as $\beta^{*}$. The choice data for each design were then analyzed using an MNL model, and the corresponding parameter estimates were recorded. To ensure the robustness of our results, we conducted 500 independent simulations, generating 500 datasets and the associated parameter estimates for each design.

To compare the accuracy of parameter estimates across different designs, we use the expected mean square error (EMSE) as a measure, which can be defined as follows:

(22) $${\text{EMSE}}_{\hat{\beta }}\left(\beta^{*}\right)=\int_{{ℛ}^{k}}{\left(\hat{\beta }-\beta^{*}\right)}^{T}\left(\hat{\beta }-\beta^{*}\right)\pi \left(\hat{\beta }\right)\;d\hat{\beta },$$

where $\pi \left(\hat{\beta }\right)$ represents the distribution of the estimates. A smaller ${\text{EMSE}}_{\hat{\beta }}\left(\beta^{*}\right)$ value indicates greater accuracy of the estimated parameters. As in [18], the EMSE value is approximated by

(23) $${\text{EMSE}}_{\hat{\beta }}\left(\beta^{*}\right)=\frac{1}{N}\sum_{n=1}^{N}{\left({\hat{\beta }}^{n}-\beta^{*}\right)}^{T}\left({\hat{\beta }}^{n}-\beta^{*}\right),$$

where N=500 is the number of simulation datasets, and ${\hat{\beta }}^{n}$ denotes the vector of estimates obtained from the *n*th simulated dataset.

Figure 1 is a boxplot presenting the square error between the parameter estimates and the true values obtained from the 500 simulation experiments for the four different designs. The black line within each box indicates the median square error for each design. It is clear from the boxplot that the main‐effects design and original design have larger medians and variances compared to the robust design and true model design. The robust design and true model design have similar performance, with the true model design having a slightly lower median.

**FIGURE 1:**
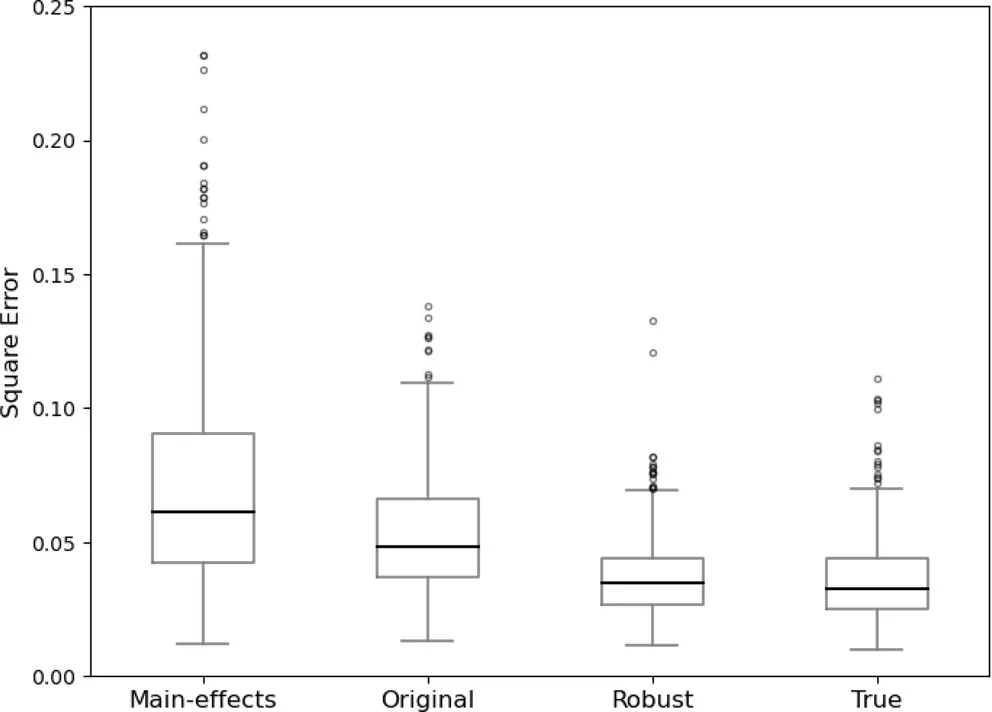
Square error of the estimates obtained from different partial profile designs in each simulation.

The specific EMSE values for the four designs are presented in Table 6. The EMSE of the main‐effects design is nearly twice that of the true model design, while the original design's EMSE increases by about 50%. This indicates that the accuracy of parameter estimates is significantly reduced for these two designs in the face of model misspecification. In contrast, the EMSE of the robust design is close to that of the true model design, further validating that SA model‐robust design performs relatively well across different underlying models.

**TABLE 6:** EMSE values comparing estimation accuracy of four different designs.

	Main‐effects	Original	Robust	True model
EMSE	0.0714	0.0528	0.0370	0.0361

## 8 Conclusion and Discussion

This paper introduces an integrated SA algorithm for constructing Bayesian optimal partial profile interaction‐effects designs. Our SA algorithm starts with an initial random partial profile design. In each iteration, it randomly selects an attribute from a random profile within a choice set, then determines whether the attribute is constant, and generates corresponding random perturbations. Our exploration rule ensures that each new design generated during each iteration satisfies the constraint that each choice set in the partial profile design contains a certain number of constant attributes. Compared to other algorithms focused on partial profile interaction‐effects designs, our algorithm can handle any number of profiles and does not require the unrealistic assumption that respondents have no specific preference among the profiles. Compared to the commonly used two‐stage CE algorithm, our SA algorithm takes into account the impact of interaction effects on the distribution of constant attributes and can flexibly optimize both the distribution of constant attributes and attribute levels simultaneously. Through extensive computational experiments, we have demonstrated that our SA algorithm consistently outperforms the CE algorithm in generating more efficient designs across all DCE scenarios that we have considered. This advantage is particularly pronounced when the prior variance is large and when the model includes a greater number of interaction terms.

Since information about interaction effects is often limited during the experimental design stage, we suggest a model‐robust design strategy to achieve good parameter estimate accuracy regardless of the underlying model. Our robust design employs a composite optimality criterion that accounts for both the main‐effects model and the interaction‐effects model. Through simulation experiments and a real‐life healthcare case study, we demonstrate that our robust design performs well under various cases of model misspecification.

From a practical perspective, incorporating interaction effects into the model significantly increases the number of parameters, while the attribute overlap in partial profile designs generally reduces statistical efficiency. Consequently, obtaining estimates with the same level of precision as a full‐profile design often requires a greater number of choice sets. In such cases, rather than relying on a single design, we recommend generating a larger set of choice sets and distributing them across multiple survey groups. This approach allows for the collection of more statistical information while preventing individual respondents from being overwhelmed by an excessive number of choice tasks.

Future research could extend our work by developing an adaptive partial profile design approach. In most applications of partial profile designs, the number of constant attributes within each choice set remains fixed. However, in practice, as respondents progress through the experiment, they may experience fatigue and boredom, which can in turn introduce order effects and reduce attentiveness [49, 50]. In such cases, the likelihood of respondents resorting to non‐compensatory decision rules may significantly increase. To deal with this, future studies could explore adaptive partial profile designs where the number of constant attributes varies based on the position of the choice set within the experiment. Specifically, in the earlier choice sets, where respondents' attention is relatively high, fewer attributes could be held constant. Conversely, in later choice sets, where respondent fatigue is more pronounced, increasing the number of fixed attributes could help alleviate cognitive burden and improve response quality.

Several limitations should be noted. First, although the main comparative simulation study covers a broad range of design scenarios, its conclusions regarding the relative performance of SA and CE are obtained under a specific set of experimental conditions, including 24 choice sets, six attributes, two or three profiles per choice set, and the particular prior distributions and interaction structures considered. Appendix D extends the computational assessment to designs with larger numbers of profiles and attributes, but it does not establish that the relative efficiency gains observed in Section 5 will persist unchanged in substantially larger or structurally different DCEs. The comparative results should therefore be interpreted within the range of settings examined rather than as a guarantee of equivalent gains across all possible partial profile design problems.

A further limitation concerns the behavioral assumptions underlying the design criterion. Our framework assumes that respondents attend to the levels of attributes held constant within a choice set, since these levels enter the utility function and contribute to the identification of interaction effects. Existing evidence on level overlap suggests that partial profile designs can reduce task complexity and improve attention to varying attributes [10, 11], but respondents may devote less attention to constant attributes or exhibit attribute non‐attendance when those attributes provide no within‐set contrast. It therefore remains unclear whether respondents process the levels of attributes held constant within a choice set in the same way, or to the same extent, as the levels of attributes that vary. If constant and varying attributes are processed differently, the statistical information anticipated at the design stage may not be fully realized in observed choice behavior. The current framework does not explicitly model such differences in attribute processing. Future work could therefore examine how respondents process constant attributes and whether such behavioral responses should be incorporated into the design criterion.

## Funding

The authors have nothing to report.

## Conflicts of Interest

The authors declare no conflicts of interest.

## Figures and Tables

**FIGURE A1:**
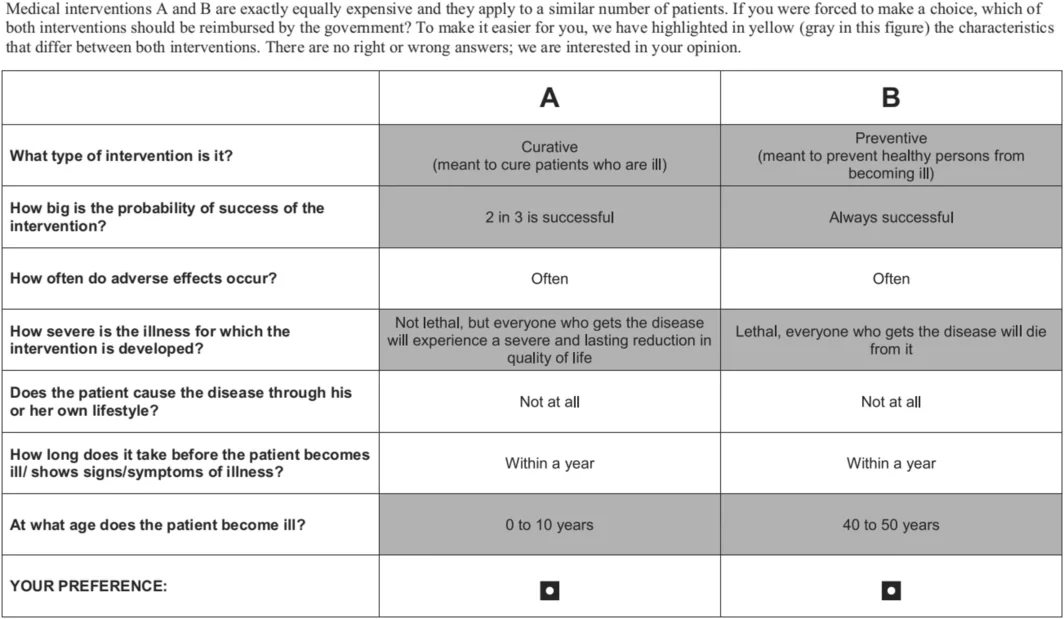
Example of a choice set used in the healthcare intervention DCE.

**FIGURE C1:**
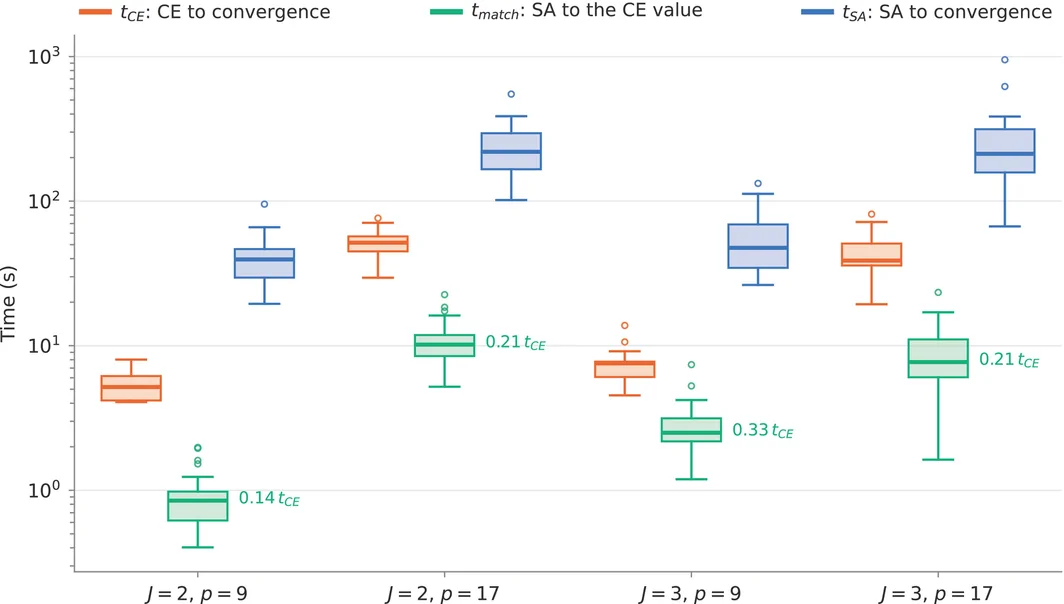
Paired run‐time comparison over 30 starting designs in each setting. For each pair, $t_{\mathrm{CE}}$ is the CE convergence time, $t_{\text{match}}$ is the first time at which SA attains the final CE criterion value, and $t_{\mathrm{SA}}$ is the SA convergence time. Numbers beside the middle boxes report the median $t_{\text{match}}/t_{\mathrm{CE}}$ ratio. The vertical axis is logarithmic.

**FIGURE D1:**
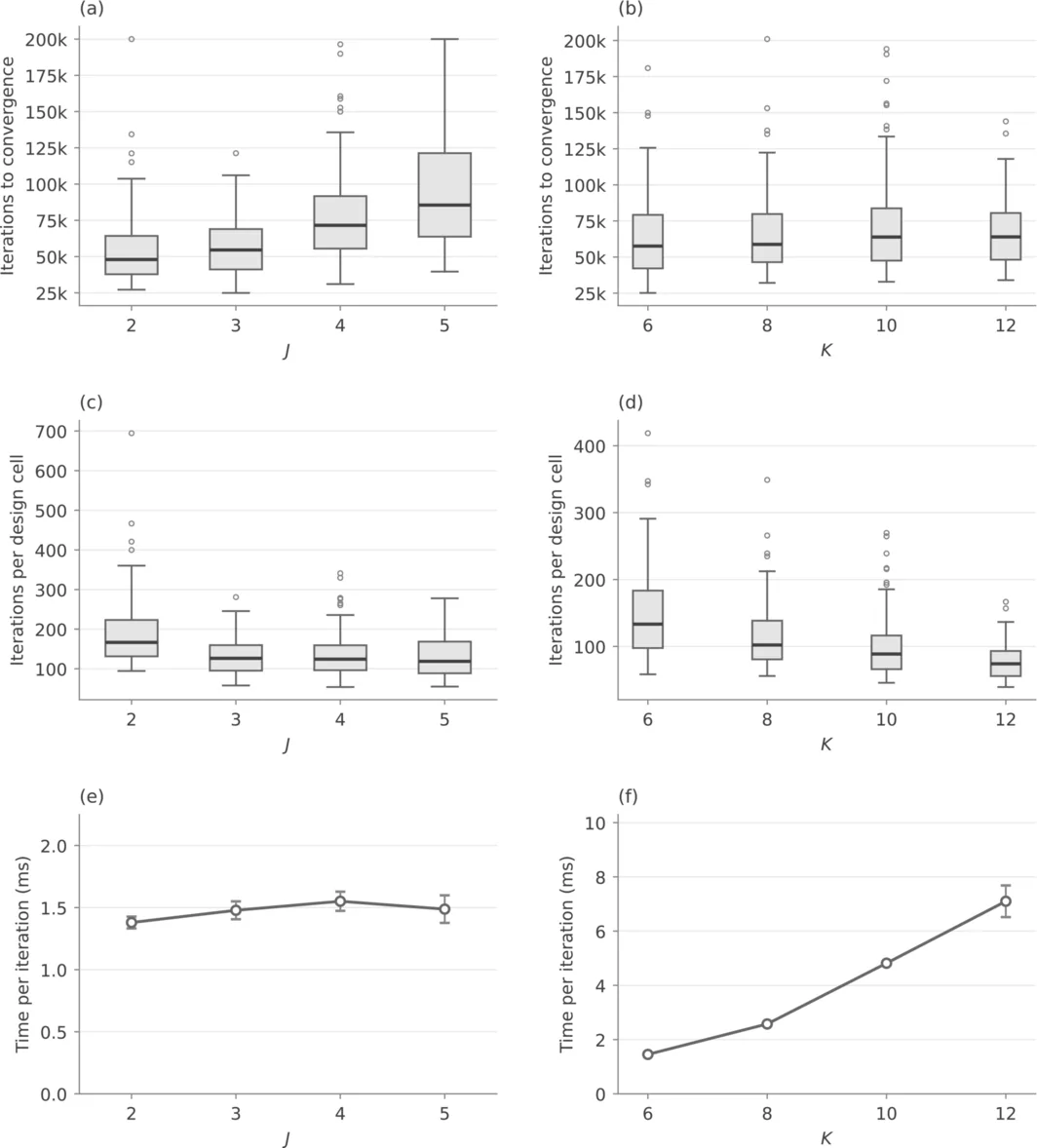
Scaling behavior of the SA algorithm over 100 independent starting designs per setting. The left column varies the number of profiles per choice set J with six attributes; the right column varies the number of attributes K with three profiles per choice set. Panels (a, b) show iterations to convergence, panels (c, d) show iterations per design cell, and panels (e, f) show mean time per iteration.

## Data Availability

The MATLAB implementation of the proposed SA algorithm, together with the scripts that reproduce all simulation experiments and the designs reported in this paper, is publicly available at https://github.com/YichengMao98/partial_profile.

## Appendix A Detailed Attribute Levels and Experimental Design of the Healthcare Intervention Discrete Choice Experiment

**TABLE A1:** Attributes and levels for the healthcare intervention DCE.

Attribute	Level
$x_{1}$: What type of intervention is it?	1. Preventive (aiming to prevent healthy persons from becoming ill)
	2. Curative (aiming to cure people who are ill)
$x_{2}$: How big is the probability of success of the intervention?	1. 1 in 3 is successful (33%)
	2. 2 in 3 is successful (66%)
	3. Always successful (100%)
$x_{3}$: How often do adverse effects occur?	1. Often
	2. Rarely
	3. Never
$x_{4}$: How severe is the illness for which the intervention is developed?	1. Not lethal, but everyone who gets the disease will experience a short period of illness without lasting effects (not severe)
	2. Not lethal, but everyone who gets the disease will experience a severe and lasting reduction in quality of life (severe)
	3. Lethal, everyone who gets the disease will die from it (lethal)
$x_{5}$: Does the patient cause the disease through his or her own lifestyle?	1. Fully
	2. Partly
	3. Not at all
$x_{6}$: How long does it take before the patient becomes ill/shows signs/symptoms of illness?	1. After 20 years
	2. After 5 years
	3. Within a year
$x_{7}$: At what age does the patient become ill?	1. 80–90 years
	2. 60–70 years
	3. 40–50 years
	4. 20–30 years
	5. 0–10 years

**TABLE A2:** The original partial profile designs used in the healthcare intervention DCE.

	Survey 1							Survey 2							Survey 3						
Choice set	1	2	3	4	5	6	7	1	2	3	4	5	6	7	1	2	3	4	5	6	7
1	2	3	1	2	1	3	5	2	3	2	2	3	2	3	2	3	2	1	1	3	4
1	2	3	2	1	1	2	3	2	3	2	3	2	1	4	2	3	2	3	2	2	2
2	2	3	3	2	1	2	1	2	3	3	2	2	3	1	2	3	3	3	1	3	2
2	2	3	1	1	3	3	1	2	3	2	3	3	1	1	2	3	2	2	2	2	2
3	1	2	3	3	3	1	3	1	3	2	1	3	3	1	1	1	1	1	2	2	1
3	1	3	3	3	2	3	2	1	2	2	2	3	1	2	1	3	1	1	1	1	2
4	1	2	2	1	1	1	4	1	2	2	1	1	3	5	1	3	2	3	1	1	1
4	1	1	2	2	2	1	2	1	1	1	1	3	3	4	1	2	1	3	1	2	3
5	1	3	2	2	2	2	3	1	1	2	2	3	2	1	1	2	3	1	3	3	2
5	1	1	1	2	2	3	5	1	2	3	2	2	1	1	1	3	1	3	3	3	5
6	1	1	3	3	1	3	3	1	1	2	1	1	2	4	1	1	3	3	2	1	4
6	1	2	1	1	2	3	3	1	2	3	2	1	2	1	1	3	1	2	1	1	4
7	2	3	1	3	1	2	2	1	3	1	2	3	3	5	1	3	2	2	2	2	3
7	1	3	1	3	2	3	3	2	3	1	3	2	3	3	2	3	2	3	1	2	4
8	1	3	3	2	2	1	4	2	3	3	2	3	1	1	2	3	1	2	1	1	3
8	2	3	3	1	3	1	2	1	3	2	2	3	3	3	1	3	3	2	1	2	4
9	2	3	1	1	2	2	2	2	3	2	1	2	3	5	2	3	2	3	1	3	1
9	1	3	3	1	3	1	2	1	3	3	1	3	3	3	1	3	1	3	3	1	1
10	2	3	2	2	1	3	1	1	3	1	3	2	2	1	1	3	3	1	2	3	5
10	1	3	3	1	1	3	5	2	3	3	1	2	1	1	2	3	2	2	2	3	4
11	2	3	1	2	3	2	4	1	2	2	2	1	3	1	2	3	2	3	1	3	5
11	1	2	1	3	3	3	4	2	3	2	2	1	1	2	1	2	2	3	2	3	4
12	1	2	2	3	3	2	2	1	2	3	3	1	3	3	1	2	1	3	1	2	2
12	2	3	2	1	3	3	2	2	3	3	3	2	1	3	2	3	1	2	1	1	2
13	2	3	1	2	2	3	1	1	1	3	2	3	2	4	2	3	1	1	1	2	1
13	1	2	2	2	2	3	4	2	3	3	3	1	2	4	1	1	1	2	1	3	1
14	1	2	2	1	3	3	5	1	2	3	1	3	2	1	1	1	2	1	2	1	3
14	2	3	3	1	1	3	5	2	3	1	3	3	2	1	2	3	1	1	1	1	3

## Appendix B Comparison of Partial Profile Designs for the Healthcare Intervention Discrete Choice Experiment

**TABLE B1:** Three different partial profile designs using different strategies for the healthcare intervention DCE.

		Main‐effects							Robust							True model						
Survey	Choice set	1	2	3	4	5	6	7	1	2	3	4	5	6	7	1	2	3	4	5	6	7
1	1	2	2	1	2	3	3	1	1	1	3	2	2	2	1	2	3	2	1	2	3	2
1	1	1	2	3	2	1	3	3	1	3	3	1	2	3	3	2	3	3	2	1	3	4
1	2	1	3	1	3	2	3	3	1	3	2	3	2	1	1	2	2	1	3	1	3	3
1	2	2	1	1	3	1	3	4	1	1	1	3	2	3	4	2	2	3	1	3	3	4
1	3	1	3	3	1	2	3	5	2	3	2	3	2	3	2	1	3	3	1	2	3	3
1	3	2	3	3	2	1	3	3	1	3	3	2	2	3	5	1	1	3	3	2	1	5
1	4	1	2	3	1	1	3	4	2	2	2	3	2	3	2	1	1	2	1	2	1	4
1	4	1	1	3	2	1	2	5	1	2	2	1	3	3	4	1	1	1	3	2	3	1
1	5	1	2	1	3	3	2	3	1	3	2	3	2	2	4	1	2	3	1	2	1	4
1	5	1	1	2	2	3	3	3	1	2	1	3	2	3	3	1	2	2	1	1	2	2
1	6	1	3	2	3	3	1	2	2	3	1	2	3	3	3	2	1	3	2	1	3	2
1	6	1	3	1	1	3	2	4	1	2	3	3	3	3	3	2	1	1	3	2	3	1
1	7	1	2	3	2	2	3	3	2	1	2	2	1	3	2	2	2	2	3	1	3	3
1	7	1	3	3	1	2	1	5	2	3	3	1	1	3	3	1	1	3	3	2	3	3
1	8	2	2	3	3	3	3	1	1	2	1	2	2	3	2	2	1	3	2	1	3	5
1	8	2	3	3	2	2	3	5	2	2	1	1	1	3	3	1	2	3	2	2	2	5
1	9	1	2	1	2	3	1	1	2	1	3	2	2	3	4	2	3	3	3	1	3	1
1	9	1	1	3	1	3	1	2	2	2	3	1	3	3	5	1	1	3	3	2	3	5
1	10	1	1	3	2	2	1	5	1	1	3	3	3	1	5	1	1	3	1	1	2	1
1	10	1	3	3	1	2	3	3	1	2	3	3	2	3	4	1	1	1	2	1	1	4
1	11	1	1	3	2	1	3	4	1	2	2	2	1	2	3	1	3	3	3	1	2	5
1	11	2	1	2	3	1	3	3	1	2	3	1	1	1	4	2	2	3	3	2	3	5
1	12	1	2	3	1	2	3	5	2	2	2	1	2	3	4	2	1	3	1	3	3	3
1	12	2	3	3	1	1	3	1	2	2	1	3	1	3	1	2	1	1	2	2	3	2
1	13	1	3	3	1	3	1	3	1	1	1	1	2	2	1	2	3	3	1	2	3	4
1	13	1	3	2	1	2	3	2	1	1	1	2	1	1	4	2	1	3	2	3	3	5
1	14	1	2	2	2	1	3	4	1	1	3	1	1	3	3	2	3	3	2	3	3	2
1	14	1	2	1	1	2	3	3	2	3	1	1	1	3	1	2	1	3	3	2	3	5
2	1	1	2	1	2	3	1	2	1	2	1	3	3	1	2	1	2	2	3	2	3	4
2	1	1	2	3	3	2	1	1	1	2	3	2	3	2	1	2	1	1	3	3	3	4
2	2	1	2	3	3	1	2	2	2	3	2	1	1	3	4	1	3	2	3	2	1	1
2	2	1	1	3	3	2	3	5	2	3	3	2	2	3	3	1	1	2	2	2	2	2
2	3	1	1	3	3	3	3	1	1	3	1	2	1	2	3	1	1	3	2	3	3	4
2	3	1	3	3	3	1	1	5	1	2	3	2	1	3	4	2	2	1	2	2	3	4
2	4	1	1	2	1	3	3	4	2	1	3	3	1	3	2	2	2	3	1	1	3	5
2	4	2	1	3	1	2	3	5	2	3	1	3	2	3	4	2	1	2	3	1	3	4
2	5	1	2	3	1	3	1	1	2	1	1	1	3	3	3	1	1	1	3	3	1	1
2	5	1	2	1	1	1	2	3	2	3	1	2	1	3	1	1	1	3	1	3	2	5
2	6	1	1	2	3	3	3	2	2	2	2	2	2	3	1	1	3	3	1	3	2	3
2	6	1	1	3	2	3	2	1	2	3	3	2	1	3	2	1	1	3	2	3	3	1
2	7	1	1	3	3	1	3	5	2	2	1	3	1	3	3	1	2	2	1	2	3	3
2	7	2	1	3	2	2	3	2	2	2	3	2	3	3	1	1	3	1	1	2	2	2
2	8	2	1	3	3	1	3	2	1	1	2	1	1	3	1	2	1	2	3	2	3	2
2	8	2	2	3	2	2	3	3	1	1	3	1	2	1	3	1	2	1	3	3	3	2
2	9	1	1	1	1	2	3	3	2	2	1	2	1	3	2	1	2	3	2	2	2	2
2	9	1	2	3	1	2	2	2	2	1	2	3	1	3	1	1	2	3	1	1	3	5
2	10	2	1	1	1	1	3	4	1	1	2	1	2	3	2	1	1	3	3	3	2	3
2	10	1	2	2	1	1	3	1	1	1	1	3	2	2	4	1	3	3	1	3	1	5
2	11	1	3	1	3	1	2	2	2	1	1	2	2	3	2	1	2	1	1	3	2	4
2	11	1	1	3	3	1	1	3	2	2	2	2	1	3	4	1	2	3	2	3	1	2
2	12	2	1	1	3	3	3	1	1	3	3	2	1	3	2	2	3	3	1	1	3	5
2	12	2	2	3	1	3	3	4	2	1	2	2	3	3	2	2	2	3	2	2	3	3
2	13	1	3	3	2	3	3	3	1	1	3	3	1	3	2	1	3	2	1	3	3	1
2	13	2	2	3	2	2	3	1	1	1	2	3	2	1	4	2	2	3	1	1	3	1
2	14	1	2	1	3	3	3	1	2	2	2	3	2	3	2	1	2	1	3	1	3	4
2	14	2	2	2	1	3	3	3	2	1	1	3	3	3	4	1	2	1	2	3	1	3
3	1	1	2	2	2	2	2	4	1	2	2	1	1	2	2	1	2	1	1	3	3	1
3	1	1	2	3	2	3	3	2	1	3	1	1	1	3	1	2	3	2	1	1	3	1
3	2	1	2	3	3	2	1	4	2	2	3	1	2	3	1	2	3	3	2	2	3	1
3	2	1	2	3	2	3	3	5	2	1	3	2	1	3	5	2	2	3	3	1	3	4
3	3	1	1	3	2	3	2	2	2	3	1	1	3	3	2	1	3	3	2	1	2	1
3	3	1	3	1	2	3	1	4	2	2	1	2	1	3	3	1	3	2	3	1	3	2
3	4	2	3	2	1	1	3	1	1	3	3	3	1	2	1	1	2	2	2	1	3	1
3	4	2	2	1	2	1	3	2	1	3	3	2	2	1	5	2	1	1	2	3	3	1
3	5	1	1	1	1	3	2	4	1	3	2	2	2	3	3	1	3	1	3	1	3	3
3	5	1	3	1	1	1	3	1	2	2	3	2	2	3	5	1	3	2	3	3	2	1
3	6	1	2	3	2	1	1	5	2	1	3	1	2	3	5	2	1	2	2	3	3	3
3	6	1	1	3	2	2	2	2	1	1	3	3	3	3	2	2	2	1	3	3	3	2
3	7	1	2	3	3	1	3	5	2	3	2	2	1	3	3	1	3	2	2	3	3	3
3	7	1	3	3	3	3	1	2	2	2	1	3	1	3	4	2	1	3	2	2	3	3
3	8	1	1	1	3	3	2	3	1	1	3	3	2	2	4	1	1	1	2	1	3	4
3	8	1	3	2	2	3	2	1	1	2	3	3	3	3	5	1	1	2	3	1	1	3
3	9	1	3	3	2	2	3	4	1	3	1	1	3	3	1	1	1	1	2	1	2	3
3	9	2	2	3	1	2	3	5	2	1	3	1	1	3	1	1	1	1	1	3	3	2
3	10	1	3	1	1	3	3	4	1	3	3	2	3	1	1	2	3	1	1	1	3	3
3	10	1	3	2	1	1	2	3	1	3	3	1	1	2	5	2	2	2	2	1	3	1
3	11	1	1	2	3	1	2	4	1	3	3	1	3	2	4	2	3	2	1	3	3	4
3	11	1	3	1	3	1	1	2	1	3	3	3	2	1	1	2	3	3	3	1	3	2
3	12	1	1	3	1	1	3	4	2	2	3	2	3	3	3	1	3	3	2	2	3	5
3	12	1	1	2	1	3	1	3	1	2	3	3	2	3	5	1	2	3	3	2	1	4
3	13	1	2	3	3	2	1	3	1	2	2	1	1	1	3	2	3	3	2	2	3	5
3	13	1	2	3	1	3	2	5	1	1	2	1	2	3	2	2	1	3	3	3	3	1
3	14	1	2	2	3	1	1	2	1	2	1	3	3	2	2	1	3	3	1	2	3	2
3	14	1	3	3	3	1	2	1	1	1	2	3	3	3	3	2	2	1	1	3	3	2

*Note:* Gray‐shaded cells indicate constant attributes.

## Appendix C Paired Run‐Time Benchmarks of the SA and CE Algorithms

Section 5 compares the SA and CE algorithms under the same computational budget: the SA algorithm is allowed to run for the total time required by 30 CE runs. To provide a more direct run‐time comparison, this appendix pairs the two algorithms at the level of individual starting designs and records both their convergence times and the time required for SA to attain the final criterion value obtained by CE.

Four settings are used, all within the design frame of Section 5: 24 choice sets, six attributes with 2, 2, 2, 3, 3, and 3 levels, two constant attributes in every choice set, and λ=κ=1. The number of profiles per choice set is J∈{2,3}. We consider either a main‐effects model, which contains p=9 parameters under effects‐type coding, or an interaction‐effects model that additionally includes all two‐way interactions between the first attribute and each of the remaining five attributes. These interactions contribute eight additional parameters, resulting in a total of p=17 parameters.

For each setting, 30 paired runs are conducted. For each pair i, we record the time required for CE to converge, $t_{\mathrm{CE}}\left(i\right)$, the time required for SA to converge, $t_{\mathrm{SA}}\left(i\right)$, and the first time at which the incumbent SA design attains the final Bayesian 𝒟‐optimality criterion value obtained by CE, denoted $t_{\text{match}}\left(i\right)$.

CE uses its usual convergence rule, terminating when a complete pass over the design produces no further improvement. SA uses the adaptive stopping criterion described in Section 4, terminating when a complete reheating cycle produces no improvement to the incumbent best design.

Table C1 and Figure C1 summarize the 120 paired runs. SA attained the final CE criterion value in all 120 cases. The median ratio $t_{\text{match}}/t_{\mathrm{CE}}$ was 0.143, 0.210, 0.335, and 0.212 across the four settings. Thus, in a typical run, SA reached the design quality ultimately obtained by CE after approximately 14%–34% of the time required for CE to converge.

SA continued to improve after matching the CE solution. The mean ${𝒟}_{B}$‐efficiency of the CE design relative to the final SA design was 0.885, 0.812, 0.916, and 0.808 in the four settings. The difference was larger in the interaction‐effects settings, consistent with the results of Section 5.

Running SA to its own adaptive convergence criterion required more time than a single CE run, with mean convergence times ranging from 39.6 to 258.2 s. However, these times remained well below the computational budget used for the main comparison in Section 5, which corresponds to the combined runtime of 30 CE starts.

These paired benchmarks distinguish between the time required to obtain a design of comparable quality and the time required for each algorithm to satisfy its own stopping criterion. Although SA generally runs longer than a single CE start when allowed to converge fully, it reaches the final design quality obtained by CE substantially earlier in the search and subsequently continues to improve the design. This explains why SA performs favorably under the equal‐computational‐budget comparison used in Section 5.

**TABLE C1:** Paired run‐time comparison of the CE and SA algorithms over 30 starting designs in each of four settings.

J	p	$t_{\mathrm{CE}}$	$t_{\text{match}}$	$t_{\mathrm{SA}}$	$t_{\text{match}}/t_{\mathrm{CE}}$	${𝒟}_{B}$‐eff of CE
2	9	5.4 (1.3)	0.9 (0.4)	39.6 (15.2)	0.143	0.885
2	17	51.3 (11.7)	11.0 (4.0)	235.8 (99.2)	0.210	0.812
3	9	7.2 (2.1)	2.8 (1.2)	55.1 (25.5)	0.335	0.916
3	17	42.8 (14.1)	8.8 (4.6)	258.2 (172.1)	0.212	0.808

*Note:* Times are in seconds with standard deviations in parentheses. $t_{\text{match}}$ denotes the first time at which the incumbent SA design attains the final criterion value obtained by CE. The ratio column reports the median pairwise value of $t_{\text{match}}/t_{\mathrm{CE}}$, and the final column gives the mean ${𝒟}_{B}$‐efficiency of the CE design relative to the final SA design.

## Appendix D Scalability of the Simulated Annealing Algorithm

The computational cost of an SA run can be decomposed into the number of iterations required for convergence and the cost of evaluating each iteration:

(D1)
total time=(iterations required)×(costperiteration).

We examine these two components under two forms of increasing design complexity: increasing the number of profiles per choice set and increasing the number of attributes.

### Cost Per Iteration

Let p denote the number of parameters in the model. The Bayesian criterion in Equation (13) is evaluated using the spherical‐radial quadrature rule of [43], with

(D2)
Q(p)=1+2(p+1)(p+2)

quadrature points.

Because each SA iteration modifies only one choice set, the corresponding information matrix can be updated locally. The computational cost per iteration is therefore approximately

(D3)
$$Q\left(p\right)\left[𝒪\left({\mathrm{Jp}}^{2}\right)+𝒪\left(p^{3}\right)\right],$$

where J is the number of profiles per choice set. This expression suggests that increasing J should have a relatively modest effect on the cost of one iteration, whereas increasing the number of attributes can have a stronger effect because it increases the parameter dimension p and hence the number of quadrature points.

### Scaling With the Number of Profiles

We first vary the number of profiles per choice set over J∈{2,3,4,5} while holding the remaining design characteristics fixed. The experiment uses 24 choice sets and six attributes with 2, 2, 2, 3, 3, and 3 levels, with two attributes held constant in each choice set. Effects‐type coding is used for all attributes, yielding nine main‐effect parameters. We additionally include two‐way interactions between Attribute 1 and Attribute 2, and between Attribute 1 and Attribute 4, contributing three interaction parameters in total. The resulting model therefore contains p=12 parameters. For each value of J, the SA algorithm is run from 100 independent random starting designs using the same adaptive stopping criterion described in Section 4.

Panel A of Table D1 shows that the mean cost per iteration remains nearly unchanged as J increases, ranging from 1.380 to 1.551 ms. The number of iterations increases with the size of the design, but substantially less than proportionally: increasing J from 2 to 5 enlarges the number of design cells by a factor of 2.5, while mean wall‐clock time increases from 76.8 to 141.2 s, a factor of 1.84. These results indicate that the SA algorithm can accommodate choice sets with more than two profiles without a comparable increase in computational cost.

### Scaling With the Number of Attributes

We next fix the number of profiles at J=3 and vary the number of attributes over K∈{6,8,10,12}. The attributes are divided equally between two‐level and three‐level attributes, and half are held constant in each choice set. The model contains the same interaction structure across settings, so the number of parameters increases from 12 to 21 as K increases. Again, 100 independent SA runs are performed for each setting.

Panel B of Table D1 shows that increasing the number of attributes has little effect on the number of iterations required for convergence. Instead, the increase in total runtime is driven primarily by the higher cost of evaluating each iteration. Mean time per iteration rises from 1.453 ms at K=6 to 7.100 ms at K=12, while the mean number of iterations remains of the same order across all four settings. Correspondingly, mean wall‐clock time rises from 94.7 to 471.9 s.

### Discussion

The two experiments show that the SA algorithm scales differently with the two main dimensions of design size. Increasing the number of profiles per choice set mainly increases the number of design elements that must be searched, while having little effect on the cost of evaluating each iteration. Increasing the number of attributes primarily increases the cost of each criterion evaluation through the larger parameter dimension and the larger number of quadrature points.

Within the ranges considered here, neither form of enlargement leads to an excessive increase in computational cost. Designs with up to five profiles per choice set and twelve attributes remained computationally tractable, with mean runtimes below 8 min in all settings examined. These results provide numerical evidence that the proposed SA algorithm is not restricted to paired choice sets or to the six‐attribute setting used in the main simulation study. Nevertheless, substantially larger designs may involve higher computational costs, particularly as the number of parameters and interaction terms increases (Figure D1).

**TABLE D1:** Behavior of the SA algorithm as the design is enlarged, over 100 independent random starting designs per setting.

				Iterations			Time (s)		
	Design cells	p	Q(p)	Mean (SD)	Median	Iterations per cell	Mean (SD)	Median	ms per iteration
(A) Number of profiles per choice set J									
2	288	12	365	55 547 (25933)	47 975	192.9	76.8 (36.8)	64.8	1.380
3	432	12	365	57 116 (20925)	54 574	132.2	84.2 (30.4)	80.2	1.478
4	576	12	365	77 999 (32479)	71 563	135.4	120.9 (50.0)	111.2	1.551
5	720	12	365	94 935 (38556)	85 496	131.9	141.2 (58.6)	130.2	1.487
(B) Number of attributes K									
6	432	12	365	65 155 (28718)	57 594	150.8	94.7 (41.9)	82.6	1.453
8	576	15	545	67 019 (28897)	58 824	116.4	172.9 (75.2)	151.8	2.576
10	720	18	761	71 989 (32985)	63 874	100.0	346.8 (159.1)	306.7	4.816
12	864	21	1013	66 666 (23282)	63 976	77.2	471.9 (166.2)	438.4	7.100

*Note:* Panel A varies the number of profiles per choice set with six attributes; Panel B varies the number of attributes with three profiles per choice set. Standard deviations are given in parentheses.
